# Protein engineering of the aldoxime dehydratase from *Bacillus* sp. OxB-1 based on a rational sequence alignment approach

**DOI:** 10.1038/s41598-021-92749-0

**Published:** 2021-07-12

**Authors:** Keiko Oike, Jens Sproß, Daisuke Matsui, Yasuhisa Asano, Harald Gröger

**Affiliations:** 1 Chair of Industrial Organic Chemistry and Biotechnology, Faculty of Chemistry, Bielefeld University, Universitätsstraße 25, 33615 Bielefeld, Germany; 2 Biotechnology Research Center and Department of Biotechnology, Toyama Prefectural University, 5180 Kurokawa, Imizu, Toyama 939-0398 Japan

**Keywords:** Biocatalysis, Biocatalysis, Enzymes

## Abstract

Recently, the program INTMSAlign_HiSol for identifying aggregation hotspots in proteins only requiring secondary structure data was introduced. We explored the utility of this program further and applied it for engineering of the aldoxime dehydratase from *Bacillus* sp. OxB-1. Towards this end, the effect of inverting the hydropathy at selected positions of the amino acid sequence on the enzymatic activity was studied leading to 60% of our constructed variants, which showed improved activity. In part, this activity increase can be rationalised by an improved heme incorporation of the variants. For example, a single mutation gave a 1.8 fold increased enzymatic activity and 30% improved absolute heme incorporation.

## Introduction

**Figure 1:**
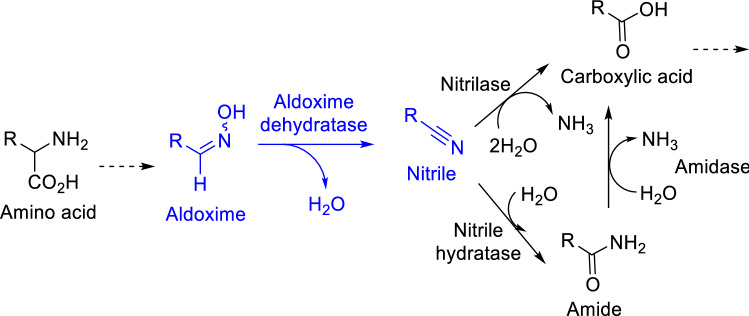
Scheme of the “aldoxime–nitrile-pathway” in microbes containing an Oxd-catalysed aldoxime dehydration step under formation of nitriles, which are further metabolised to carboxylic acids^2^.

Aldoxime dehydratases (Oxds; E.C. 4.99) are special heme containing lyases involved in the “aldoxime–nitrile-pathway” of microbes (Fig. 1). These enzymes catalyse the dehydration of aldoximes to nitriles, which are metabolised to carboxylic acids by nitrile hydratases and amidases or by nitrilases^1–4^.

The first discovered enzyme of this class is the Oxd from *Bacillus* sp. OxB-1 (OxdB), which was isolated from soil^1^. This enzyme naturally catalyses the dehydration of *E*- and *Z*-phenylacetaldoxime (*E*- and *Z*-PAOx) to phenylacetonitrile (PAN). It was purified as wild-type enzyme and expressed heterologously in *Escherichia* *coli* (*E.* *coli*)^5,6^. However, only a handful of these Oxds have been identified, isolated and characterised yet^7–13^.

Oxds can be applied for the synthesis of nitriles^13–20^ and dinitriles^21^, compound classes with diverse applications in the chemical and pharmaceutical industry^22^. Common chemical syntheses of nitriles usually require harsh reaction conditions such as the use of the highly toxic hydrogen cyanide and salts thereof or high temperature and pressure^23,24^. Therefore, Oxds are very interesting biocatalysts for substituting these reactions by a biocatalytic process under mild conditions avoiding cyanide. Furthermore, we recently showed that the aldoxime dehydration is enantioselective^25^ and that Oxds have a preference for one isomer of the aldoximes and can switch the enantioselectivity in dependency on the *E*- and *Z*-aldoxime as a substrate^26^. Chiral nitriles are of general interest as, e.g., such structural motif can be also found in drugs such as, for example, Saxagliptin^27^ and Vildagliptin^28^.

Although the enzymes show a high potential for synthetic purposes, only a few works on engineering these types of proteins are reported^29^. The recombinant Oxd from *Rhodococcus* sp. YH3-3 was targeted by directed evolution via random mutagenesis in order to improve the catalytic efficiency for the dehydration of 2-furfurylaldoxime to 2-furfuryl nitrile, a biorenewable feedstock-based nitrile with several applications in the chemical industry^29^. Other mutagenesis studies on aldoxime dehydratases so far were focused on the understanding of the substrate binding, catalytic activity and mechanism but not the improvement of catalytic properties^30–34^. The crystal structures of the Oxds from *Rhodococcus* sp. N-771 (OxdRE)^33^ and *Pseudomonas* *chloraphis* B23 (OxdA)^34^ are the only ones known up to now.

Even though the crystal structure is still unkown, OxdB is by far the most well characterised and investigated Oxd enzyme, in particular with respect to synthetic applications. However, engineering of this enzyme has not been carried out yet. Here we present a method for the improvement of the catalytic efficiency of Oxds by site-directed mutagenesis without the necessity of structural knowledge of the enzyme. We used a rational method for the calculation of these mutation sites based on multi-sequence alignment under consideration of the amino acid hydropathy.

## Results

### Assay design

First, we decided on the method being used for the engineering of the target protein which is strongly related to the library size we are able to screen. While for the dehydration of 2-furfuryl aldoxime a large library could be screened by spectroscopy, this method is not suitable for arylaliphatic aldoximes. Thus, we decided to use an already established assay monitoring the product formation of the nitrile in reversed-phase HPLC measurements according to a method developed in our previous studies^25,26^. As our intention was the identification of variants with a generally increased activity independent from a specific substrate, we performed the screening with the native substrate of OxdB, *Z*-PAOx.

### Identification of mutation sites

The program INTMSAlign was originally developed by the Asano group for the identification of novel hydroxynitrile lyases based on multi-sequence alignment^35^. In a later work, the program was modified by implementation of a tool for amino acid hydropathy calculation (INTMSAlign_HiSol)^36^. This additional tool is suitable to compare the amino acid hydropathy of each position in the target protein with the conserved hydropathy in the protein family. The difference of this hydropathy is defined as the so called HiSol score. A higher absolute value of the HiSol score indicates that opposite hydropathy is highly conserved in the selected position. This calculation method has been utilised for identification of aggregation hotspots in proteins. When the hydropathy of amino acid residues located in α-helices was inverted by exchange with highly conserved amino acids, several proteins could be solubilised for recombinant expression in *E.* *coli*^36^. We assumed that the hydropathy switch induces structural changes on the protein constitution, which can also influence the catalytic efficiency.

Toward this end, we decided to utilise the hydropathy calculation function implemented in INTMSAlign_HiSol in order to identify potential mutation sites in the sequence of OxdB. At the same time, we could limit the number of variants for our library while still finding positions with a significant difference compared to the aligned sequences. When the INTMSAlign_HiSol software was applied to the sequence of OxdB, twenty residues exhibited the highest absolute HiSol scores (Table 1). The amino acids, which are targets for the replacement by more hydrophilic residues, gave positive HiSol scores (Entries 1–10). The amino acids, which are targets for the replacement by more hydrophobic residues, gave negative HiSol scores (Entries 11–20). We did not further investigate the residues with lower absolute HiSol scores. Besides the HiSol score, the result sheet of INTMSAlign_HiSol displays the conservation rates of each amino acid in each position in the sequence of the target protein (Supplementary Information, Table S1). We chose the amino acid with the highest conservation rate for construction of variants bearing single-point mutations. The conservation of the identified residues in the gene family was spread out between low values of under 20% (Entries 8 and 11) and up to 90% (Entry 1). With this approach, we could construct our screening library by a rational approach which requires only the amino acid sequence of the protein. According to a secondary structure determination with the PSIPRED tool, the majority of the twenty identified residues with high absolute HiSol scores was located in strand (45%) or coil (30%) structures^37,38^. The other five identified residues (25%) were located in α-helices. A comparison of these mutation sites with a homology model structure of OxdB suggested that these twenty residues were not located in the active site of OxdB^32^.

**Table 1:**
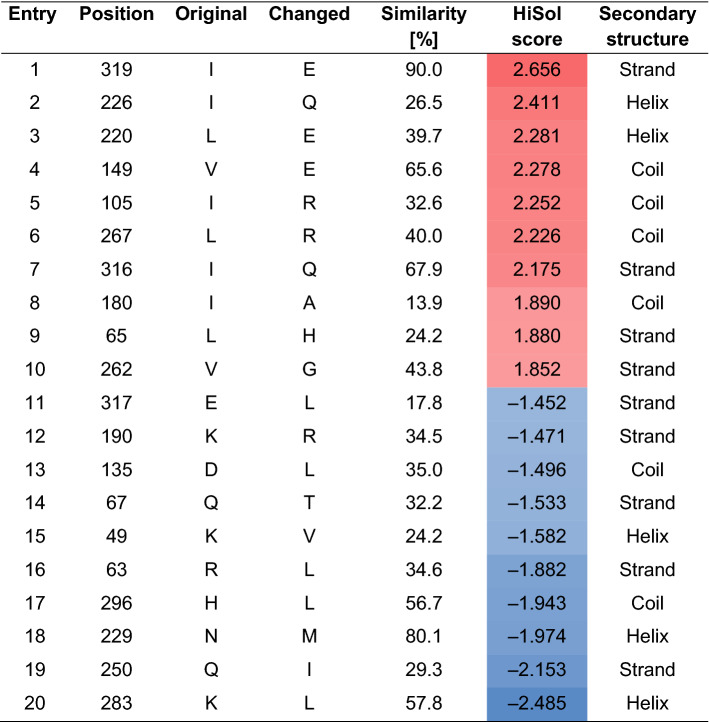
Predicted mutation sites in the sequence of OxdB by the program INTMSAlign_HiSol^36^.

The structure elements were predicted by PSIPRED^37,38^.

### Generation of the mutant library and initial screening

The identified point mutations were introduced by site-directed mutagenesis with the corresponding primers (Supplementary Information, Table S2) and the vector pET22b harbouring the OxdB gene as template DNA, thus yielding OxdB-M1 to OxdB-M20. This vector was chosen because of its C-terminal hexahistidine-tag, which simplifies protein purification. In an initial screening, wild-type OxdB and the twenty variants were overexpressed in *E.* *coli* BL21(DE3) according to a protocol reported in literature^32^. We expressed the variants on small scale in shaking flasks. Here we wanted to make sure that the expression conditions were comparable to the actual conditions as oxygen saturation during expression of OxdB plays an important role^6^. It should be added that (in analogy to the wild-type enzyme OxdB) after expression in *E.* *coli* all of the 20 variants have been obtained in soluble form, which is illustrated by the SDS-PAGEs (see Supplementary information, Figure S2).

We then tested if the generated variants were active and compared them to the wild-type enzyme in an assay carried out with whole-cell catalysts (see Supplementary Information, Figure S1). This assay is closely related to our typical application of Oxds in catalysis. As a model substrate for this study, we chose the native substrate of OxdB, *Z*-PAOx, since it was our intention to achieve activity improvement for a substrate, which is already smoothly converted by OxdB, and no specific, “unusual” substrate with very low overall activity. In this initial screening, nearly all generated variants were active. We observed an improvement of the enzymatic activity for seven mutants. SDS-PAGE analysis (Supplementary information, Figure S2) showed that all variants were expressed in soluble form, we did not observed the formation of inclusion bodies for wild-type OxdB or any of its variants. The relative expression level compared to the wild-type enzyme was in overall slightly lower for several variants. At this stage, we did not further evaluate the whole-cell catalyst activity as we were more interested in the characterisation of the variants inter alia regarding their specific activity. In addition to the 20 variants with single point mutations, we constructed three further variants combining the most efficient variants of the initial whole-cell screening leading to promising single mutations, thus yielding Oxd-D1: L65H_I180A, OxdB-D2: I180A_K190R, and OxdB-D3: L65H_K190R.

### Activity determination of the purified OxdB variants

**Figure 2:**
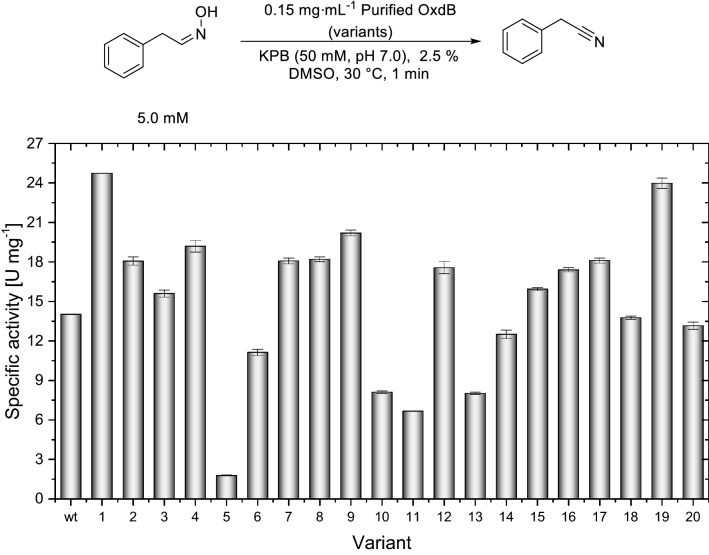
Specific activities of wild-type OxdB and its variants (purified enzyme) for the standard substrate *Z*-PAOx. The numbers of the variants refer to the mutations displayed in Table 1.

The wild-type enzyme and all 23 constructed variants were purified according to a literature known protocol by affinity chromatography on a Talon affinity resin loaded column^32^. The purity of all mutants was considered as sufficient for the subsequent experiments (see Supplementary Information, Figures S3 to S5). Again, the specific enzymatic activity was determined by measuring the initial conversion rate of *Z*-PAOx to PAN. In their purified form, a majority of 60% of the variants showed an improved specific activity compared to the wild-type enzyme (Fig. 2). The highest specific activities observed were 24.7 U mg^–1^ and 24.0 U mg^–1^ for OxdB_I319E (M1) and OxdB_Q250I (M19), respectively. These variants were no hits in the whole-cell screening, despite of their improved catalytic efficiency, which can be explained by their lower expression level (Figure S2). Five variants (OxdB-I105R (M5), V262G (M10), E317L (M11), D135L (M13)) showed activities lower than 11 U mg^–1^, corresponding to a relative activity below 79% of the wild-type enzyme. Interestingly, a different migration behaviour (slightly faster than the other proteins) was observed for OxdB-M13 in the SDS-PAGE analysis (see Supplementary Information, Figure S4). The specific activity of OxdB-M5 (I105R) was particularly low with 13% relative to the wild-type enzyme. In addition, the amount of produced protein was the lowest as well. Seven variants (OxdB_I226Q (M2), I316Q (M7), I80A (M8), K190R (M12), K49V (M15, R63L (M16), and H296L) exhibited a slightly improved activity between 17.4 and 18.2 U mg^–1^, which corresponds to a relative increase of up to 30% compared to the wild-type enzyme. OxdB_V149E (M4) and OxdB_L65G (M9) showed an activity increase of 36 and 44%, respectively. The other variants (OxdB-L220E (M3), Q67T (M14), N229M (M18), and K283L (M20)) showed no significant deviation of the specific activity compared to the wild-type enzyme (± 10%). We decided not to investigate reaction kinetics, as the *K*_m_-value for the substrate *Z*-PAOx is or can be expected to be in sub-millimolar range and, thus, a certain change of the *K*_m_-value should not alter significantly the applicability in synthetic transformations which run at significantly higher substrate concentration^5^. Furthermore, the mutations are not located in the active site.

For the variants with two combined point mutations, activities outperforming their “parents” were observed (Table 2). The double mutants OxdB_L65H_I180A (D1) and OxdB-D3_L65H_K190R (D3) showed a specific activity of 26.4 U mg^–1^ and 28.4 U mg^–1^, respectively (Entries 1 and 3). This corresponds to a 1.9- and 2.0-fold increase compared to the wild-type. It is noteworthy that the variants M8, M9 and M12 turned out not to be the most active single variants, even though they showed promising properties in the whole-cell screening. In contrast to the most active OxdB-M1 and M19 variants, however, these three variants were expressed better. Nevertheless, the observed specific activities of OxdB-D1 and OxdB-D3 were exceeding the best single mutations OxdB-I319E and OxdB-Q250I (M1). The double mutant OxdB-D2 (I180A_K190R) also showed an activity improvement, however, only exceeding its parents’ activities by less than 10% (Entry 2). The expression levels of the double variants were comparable to those of the parents. Their whole-cell activity was not tested.

**Table 2:** The specific activities of the variants OxdB-D1 to OxdB-D3 and the relative activities compared to the wild-type protein and the corresponding parent variants.

Entry	Specific activity (U mg^–1^)	Activity relative to wt (%)	Activity relative to parent 1 (%)	Activity relative to parent 2 (%)
1	26.4 ± 0.4	+ 189 ± 3	+ 31 ± 2	+ 51 ± 2
2	19.1 ± 0.2	+ 136 ± 1	+ 5.1 ± 1	+ 8.8 ± 1
3	28.4 ± 0.3	+ 203 ± 2	+ 41 ± 2	+ 62 ± 2

### Mass spectrometric analysis

**Figure 3:**
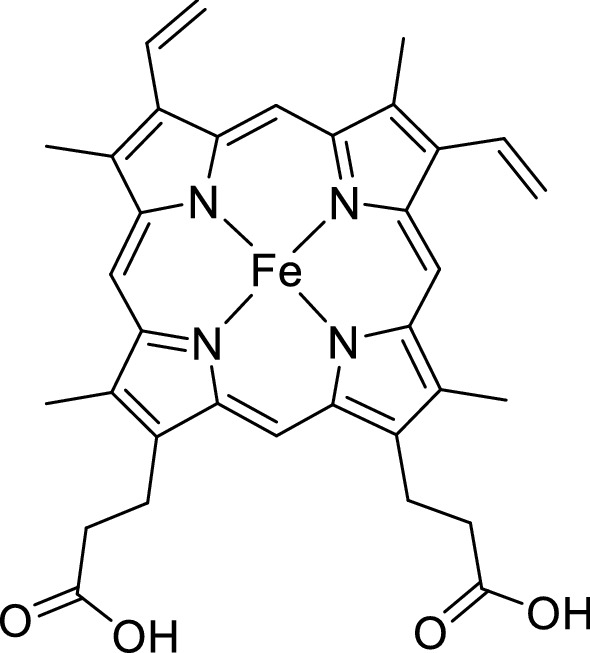
Structure of protoheme IX (heme b)^39^.

Surprisingly, a majority of the OxdB variants showed a higher activity compared to the one of the wild-type enzyme. In order to find an explanation for these observed increased activities, we speculated about the influence of the heme content of the Oxd variants on the specific activity. Oxds are dependent on the cofactor protoheme IX (Fig. 3), which is bound via coordination to a proximal histidine residue to the protein backbone^33,34^. The incorporation of the cofactor is probably necessary for the protein folding, as OxdB expressed in the absence of iron is produced exclusively in insoluble form^5,6^. Oxds typically do not contain the cofactor in all protein molecules. The heme content varies for each Oxd and is, e.g., influenced by the expression system and the position of a histidine tag^9^. For instant, OxdB typically contains 33% heme when expressed in *E.* *coli* JM109 or 35% when expressed homologously in *Bacillus* sp. OxB-1 while OxdA contains 69% heme when being expressed in *E.* *coli*. However, a rationalisation of the quantitative cofactor incorporation in Oxds has not been done until now. An influence of our mutations on the heme content might be likely as our mutation point were chosen based on a sequential alignment and similarity considerations.

**Figure 4:**
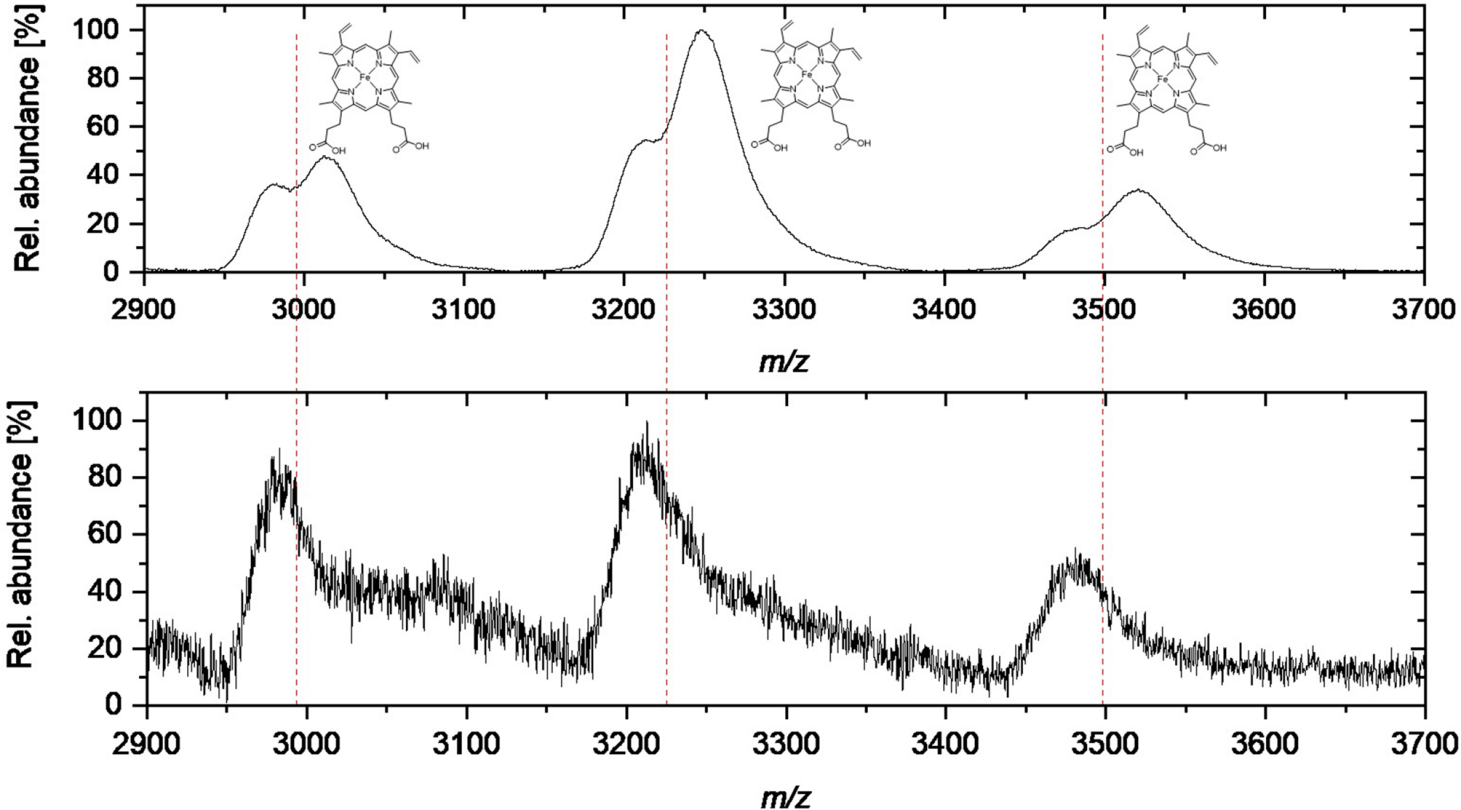
Overlay of excerpts of native (upper) and denaturated (lower) wild-type oxdb mass spectra. The shown region refers to 2930 and 3520 m/*z*, in which the peaks refering to the charge states + 11 to + 13 are displayed.

Thus, in order to gain more insight into the impact of mutations and sequence changes of Oxds on the quantitative heme content, we analysed the heme content of the wild-type OxdB and of selected variants by mass spectrometry. Toward this end, we conducted static nanoESI-MS measurements under denaturing conditions for the desired determination of the protein masses (Table 3). The following constructs were analysed: the wild-type enzyme, the most active variants (OxdB-M1, M2, M4, M8, M9, M12, M17, and M19) and OxdB-M13 because of its different gel electrophoresis behaviour (see Supplementary Information, Figure S4). All variants with two point mutations were investigated as well. Besides the charge state envelope of the protein ions, protoheme IX was identified with a high mass accuracy (*m*/*z* 616.1773, *m*/*z*_*theo*_ 616.1774, Δmass 0.1 mmu; deviation: 0.17 ppm) in measurements under denaturing conditions. This confirmed that the heme cofactor is bound non-covalently to the enzyme as previously reported^5,31^. Under native conditions, the static nanoESI-MS spectra contained a charge state envelope at significantly higher *m*/*z* values with a lower amount of charge states (Fig. 4). For most variants, each charge state consisted of two peaks with a typical mass shift of 615 ± 40 Da (after charge state clean up). This is in good agreement for two enzyme species, one with and the other without the cofactor protoheme IX.

**Table 3:** Protein masses determined by static nanoESI-MS for wild-type OxdB and selected variants.

Variant	M_sequence,_ _theo_ (Da)	M_Denatured_, _exp_ (Da)	ΔMass to sequence (Da)	M_Native,_ _exp_ (Da)	ΔMass to denatured protein (Da)
Wild-type	41,216.78	41,255.8 ± 9.0	+ 39.02	41,901.8 ± 6.0	646.0
M1 (I319E)	41,232.73	41,248.21 ± 1.97	+ 15.45	41,863.76 ± 0.37	615.55
M2 (I226Q)	41,231.75	41,236.9 ± 4.8	+ 5.15	41,844.5 ± 2.6	607.6
M4 (V149E)	41,246.76	41,302.7 ± 6.4	+ 55.94	41,862.4 ± 2.1	559.7
M8 (I180A)	41,174.69	41,208.0 ± 6.8	+ 33.51	41,824.1 ± 6.7	615.9
M9 (L65H)	41,240.76	41,218.7 ± 4.8	– 22.06	41,829.4 ± 12.4	610.7
M12 (K190R)	41,244.79	41,291.3 ± 4.6	+ 46.51	41,863.8 ± 0.4	572.5
M13 (D135L)	41,214.85	41,216.4 ± 0.7	+ 1.55	41,830.60 ± 0.04	615.0
M17 (H296L)	41,192.79	41,229.0 ± 8.0	+ 36.21	41,791.2 ± 3.3	562.2
M19 (Q250I)	41,201.80	41,182.9 ± 7.8	– 18.90	41,801.6 ± 1.0	618.7
D1 (L65H_I180A)	41,198.68	41,215.9 ± 2.6	+ 17.22	41,829.9 ± 0.4	614.0
D2 (I180A_K190R)	41,202.71	41,217.82 ± 0.16	+ 15.09	41,824.02 ± 0.91	606.2
D3 (L65H_K190R)	41,268.77	41,298.9 ± 1.1	+ 30.13	41,889.8 ± 18.8	590.8

The protein masses after charge state clean-up in the denatured state and the native state are given in Da. For the native state, two masses were determined, only the result with the higher molecular mass is displayed in this table. For the original data see the processed spectra displayed in Figures S7–S19.

The determined molecular masses of wild-type OxdB and its variants exhibit deviations compared to the theoretical molecular weight calculated from the sequence, which exceeded the usual error range of such an analysis. These differences can be explained by residual amounts of salt, which lead to broad signals in the mass spectrum, causing the inaccuracy of the molecular masses. It should be added that these effects are quite common in measurements under native conditions as ions of the buffer (here: ammonium acetate) form salt adducts with the protein. However, in the case of denaturing conditions protein clean-up might not be sufficient. Interestingly, the variants OxdB-M9 and OxdB-M19 yielded a lower protein mass compared to the theoretical mass derived from the sequence. An explanation for this finding is difficult without further data.

**Figure 5:**
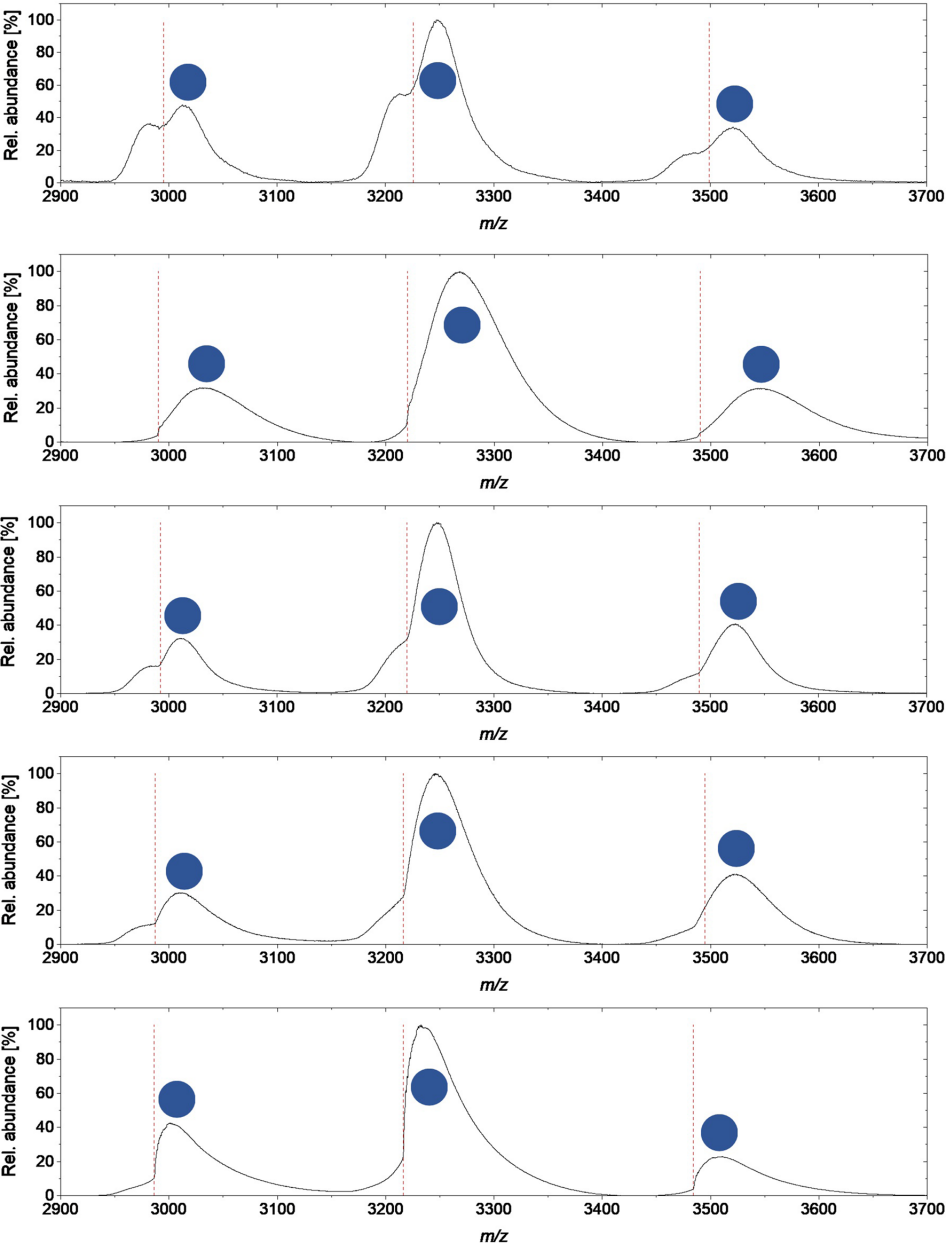
Excerpt of native mass spectra of wild-type OxdB and selected variants (from top to bottom: wild-type OxdB, OxdB_I319E (M1), OxdB_L65H (M9), OxdB_Q250I (M19), OxdB_L65H_I180A (D1). The shown region refers to 2930 and 3520 m/*z*, in which the peaks referring to the charge states + 11 to + 13 are displayed. The dashed red lines indicate the separation of the peaks of apoprotein and holoprotein. The blue dots indicate the peaks of the holoprotein.

Nevertheless, we were pleased to find that by overlaying both spectra of the denatured and the native protein (or an excerpt of a few charge states) the protein species with bound heme can be easily differentiated from the protein species without heme. An example for this approach is visualised for the wild-type enzyme (Fig. 4). By comparing the peak intensities of the protein signals of apo- and holoprotein in the native mass spectra, a rough estimate about the relative heme content can be made (Fig. 4). For the wild-type enzyme, the ratio of the integrals of the peaks of apoprotein to holoprotein was estimated to be 30:70, thus referring to 70% holoprotein formed. For this analysis method, we assumed that the relative ionization efficiency of the different protein species is not altered by the presence of the cofactor. For all variants investigated via native MS measurements, differences in the ratio of the relative peak integrals of a selected charge state were observed (e.g., Fig. 5). For all mutants with an improved specific activity, we hypothesised that the amount of heme incorporated during the expression had been increased.

### Correlation between heme content and specific enzymatic activity

**Figure 6:**
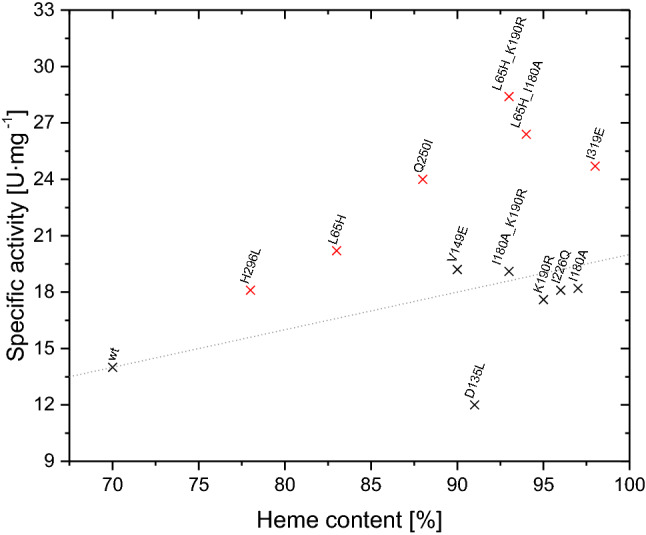
Correlation of the estimated heme content of selected OxdB variants and their specific activity. The dotted line refers to a linear dependence of the specific activity and the heme content compared to the wild-type enzyme.

We plotted the specific enzymatic activity of the wild-type enzyme and all investigated mutants against the heme content in order to visualise the correlation between these values (Fig. 6). If an increased heme content is the only reason for a higher activity, one would obtain a linear increase of the specific activity with a higher heme content. For nearly all investigated variants, a higher heme content resulted in a higher specific activity. The variant OxdB-M13 is an exception to this finding. Despite of the higher heme content of 91%, the activity was notably lower compared to wild-type OxdB.

For example, the single mutation variants OxdB-M1, OxdB-M2, OxdB-M4, OxdB-M8, OxdB-M12, and OxdB-M13, and all double mutation variants exhibited heme contents above 90%; the apoprotein was barely present in this analysis. The observed heme incorporation for these variants refers to an increase of at least 20% compared to wild-type OxdB. The specific activities of OxdB-M2, OxdB-M4, OxdB-M8 and OxdB-M12 as well as the double mutation variant OxdD2 correspond nearly ideally to a linear correlation with the heme content. Four single mutant variants (OxdB-M1, OxdB-M9, OxdB-M17 and OxdB-M19) do not correspond to the ideal linear dependence. A higher activity than expected from the heme content was observed in these cases. While the heme content of OxdB-M9 was relatively low with 83%, this variant showed the third highest activity amongst the single mutant variants. This effect was even more significant for OxdB-M19 and OxdB-M1, which were the most active single mutant variants with heme contents of 88% and 98%, respectively. For these variants, effects apart from the increased heme incorporation probably lead to the higher activity. It is very notable that the heme content could be raised from 70% to nearly total incorporation by exchanging a single amino acid. The double mutants OxdB-D1 and OxdB-D3 showed even higher catalytic efficiency than all single mutation variants. Their heme content was approximately located in between those their parents. Both variants also showed a correlation between their heme content and specific activity exceeding the linear dependence.

## Discussion

We used the algorithm of INTMSAlign_HiSol to identify mutation sites by multi-sequence alignment. The algorithm itself is a suitable tool to identify positions, in which the target protein has significantly different hydropathy and distribution compared to the consensus. By introducing highly conserved amino acid residues being available from the output file of INTMSAlign_HiSol, we constructed variants which exhibit inverted hydropathy. In addition, the high conservation of the introduced amino acids makes it more likely that the variants exhibit activity. This is in contrast to random mutagenesis-based approaches, for which the observation of inactive variants is common. The variants from our library exhibited two significant improvements compared to the wild-type protein: higher specific activity and higher cofactor incorporation. Naturally, there cannot be a direct correlation and, thus, predictability between an abstract value such as the HiSol score and higher catalytic efficiency. We assume that the likelihood of a positive influence on the protein structure is obtained by this algorithm. The method was suitable to generate small libraries by picking interesting positions without any structural knowledge about the protein apart from the amino acid sequence.

Paying respect to the deviations in the mass spectra, the heme content of the wild-type OxdB is compared to references where OxdB is expressed in *E.* *coli* JM109 harboring a pUC18 plasmid encoding the OxdB gene^6^. The wild-type OxdB as a reference for our mutagenesis showed a specific activity of 14.0 U mg^−1^ for *Z*-PAOx compared to a specific activity 8.33 U mg^−1^ reported in literature^6^. At the same time, the heme content was higher with 70% compared to 33% in literature, where the heme content was determined by precipitation with acetone/hydrochloric acid. The activity of 0.20 U mg^−1^ per % heme is lower compared to 0.25 U mg^−1^ per % heme reported in literature. This can be explained be the fusion of the hexahistidine-tag and the lower purity (single affinity chromatography vs several purification steps). The reason for the higher heme incorporation is probably the fusion of the tag, which is also reported to influence the heme content of OxdRE^9^. These considerations indicate that the MS measurements are a suitable method for estimation of the heme content and our assumption of a similar ionisation efficiency of the apoprotein and holoprotein is in good agreement with the obtained results.

With our rational approach of exchanging amino acids in residues with a hydropathy mismatch to highly conserved amino acids with the opposite hydropathy, we generated 12 mutants with an improved specific activity. The hit rate for an improved activity is therefore 60%. There are only a few strategies for improving the enzymatic activity by rational methods, especially if there is no knowledge about the structure. Typically, random mutagenesis is the method of choice for such studies^29^. However, also other multi-sequence alignments were suitable to identify mutations improving the catalytic efficiency^40^. An advantage of our method is the small library size, which is especially helpful when high-throughput screening methods are not available. Thus, the results from our study show an additional utility of the program INTMSAlign_HiSol pointing out the strength of sequence alignment. Originally, INTMSAlign_HiSol was developed for solubilisation of proteins for expression in *E.* *coli* also under higher expression temperatures. Therefore, positions located in helices are chosen for the construction of variants. In contrast to our results, a notable improvement of the catalytic efficiency was not achieved^36^. We could partly rationalise the improved catalytic efficiency of our constructed variants by showing that the absolute heme content was increased. However, we could not find improved expression levels for the majority of our variants. At the same time a major difference in our study is that we chose the already soluble protein OxdB as a target for engineering instead of insoluble proteins. It is yet to be tested if our variants show an improved expression level at higher expression temperatures. The wild-type OxdB is usually forming insoluble aggregates at this temperature. The optimised pUC18 vector encoding the OxdB gene would be more suitable for expression of our variants when considering synthetic applications^6^. The improved heme incorporation certainly represents a beneficial factor for enhanced activity besides the expression level.

As visualised in Fig. 6, some variants exceeded the specific activity expected with respect to their heme content. The three most active variants with a single mutation (OxdB-M1, OxdB-M9 and OxdB-M19) had in common that they are located in β-strand structures. In the related OxdRE, most β-strand structures form a single β-barrel consisting of eight β-strands, which also contains the catalytically active amino acid residues^33^. From the sequence alignment (Figure S6) and secondary structure determination (Table 1), we hypothesised that OxdB forms this structure element in analogy to OxdRE. β-Barrels are structures formed by hydrogen bonds of typically at least eight antiparallel β-sheets connected by loops^41^. As our mutations inverted the hydropathy of the amino acids residues, thus changing the possibility for hydrogen bond formation, a deformation of the β-barrel structure is likely. Such a deformation could relocalise the catalytically active residues contained in the β-barrel structure to a position being advantageous for substrate binding and, thus, causing a higher catalytic efficiency.

It is noteworthy that by means of introducing a single mutation, the heme content could be increased from 70% up to 98%. This is especially surprising as no mutation was located in the active site or in positions where the interaction of the protein with the heme side chains is conserved, e.g. hydrogen bond formation (see the alignment of the sequences of OxdB, OxdRE and OxdA in the Supplementary Material, Figure S6)^33,34^. Thus, it appears that another factor has an impact on the heme incorporation. In addition, we suppose that our mutations induce structural changes in the protein, which improve the interaction with the cofactor, thus, facilitating its incorporation. Still, this is a new example for another utilisation of sequence alignment as a tool for rational protein design. For comparison, there are other possibilities for improving the heme incorporation during protein expression. The most common strategy for improving the heme incorporation is the addition of the heme precursor δ-amino levulinate to the culture medium^42^, which also could be applied for Oxds. Other strain optimisation strategies use, e.g., the *E.* *coli* strain Nissle 1917, which has the natural ChuA heme receptor^43^. Alternatively, the strain *E.* *coli* RP523, which contains a porphobilinogen synthase gene disruption and a an uncharacterised permeability mutation that renders the bacteria heme-permeable, can be utilised as well^44^. All these methods, however, have in common that they require the addition of expensive heme (precursors) to the culture medium, which is a cost factor when bulk chemicals or fine chemicals should be synthesised by biocatalytic reactions. A different approach is the co-expression of a ferrochelatase, an enzyme catalysing the complexation of iron-ions into protoporphyrin IX^45^. In contrast, our method for improved heme incorporation is based on protein engineering and, thus, does not require expensive additives to the culture medium or the co-expression of another enzyme. This should be advantageous regarding the application of the enzyme. It is yet to be proved if this method also improves the heme incorporation of other Oxds and related hemoproteins or if this observation is limited to the OxdB enzyme.

## Conclusion

In summary, we engineered OxdB by a rational multi-sequence alignment approach utilising the program INTMSAlign_HiSol. It is noteworthy that 60% of our variants constructed by switching the hydropathy of amino acids showed an improved activity. For most variants, this activity correlated to an improved heme incorporation compared to the wild-type enzyme. With a single mutation, nearly full cofactor incorporation was achieved. A few variants amongst the variants with higher heme incorporation showed an improved activity not only related to the heme content. For these variants, we hypothesise that a structural change in a β-barrel structure might be related to the higher activity. We also expect that a structure elucidation of these variants will be facilitated in the future, when the crystal structure of OxdB will be solved. Such findings should then also enable a rationalisation of the beneficial impact of the individual mutations. A further task of future work is the application of the constructed variants for various synthetic purposes.

## Methods

### General information

Protein concentrations were determined according to the method from *Bradford*^46^ or using a Nanodrop One (Thermo Fisher Scientific, Massachusetts, USA). Gel electrophoresis was performed according to a protocol from *Laemmli*^47^. All chemicals used for biochemical experiments were of buffer/biochemical grade; solvents of HPLC-grade, PAOx, and PAN were self-synthesised. The protein was handled, if not stated differently, at 4 °C and stored at the same temperature. NMR-measurements were conducted on a Bruker Avance III 500HD. ^1^H-NMR measurements were performed at 500 MHz and ^13^C-NMR at 126 MHz.

### Identification of the mutation sites

The mutation sites were identified sequence of OxdB in accordance to Matsui and coworkers using the program INTMSAlign_HiSol applied to the amino acid sequence of OxdB^36^. The output file was used to calculate the HiSol score (as dimensionless value) by the following formula using the normalised hydropathy indices (*I*_*hyp*_, z-transformed) according to Kyte and Doolittle^48^

$${\text{HiSol~score~}}\left( {\text{j}} \right) = ({\text{I}}_{{{\text{hyp}}}} )_{{{\text{j}},{\text{~OxdB}}\left( {\text{i}} \right)}} - \sum\limits_{{{\text{i}} = 1}}^{{20}} {({\text{I}}_{{{\text{hyp}}}} )_{{\text{i}}} } \left\{ {({\text{R}}_{{{\text{cons}}}} )_{{{\text{ij}}}} /100} \right\}$$

The variable “*i*” represents the amino acid residues numbered in alphabetical order of their one-letter abbreviations; e.g. the *i* values of Ala, Cys and Asp are 1, 2, and 3, respectively. Thus, (*I*_*hyp*_)_*j*,OxdB(*i*)_ refers to the hydropathy index of the amino acid residue “*i*” at the *j*th residue in the sequence of OxdB. (*R*_*cons*_)_ij_ represents the appearance rate of that amino acid at the *j*th residue of OxdB in the library. We considered the amino acids with the 20 highest absolute HiSol scores as promising mutation sites.

The program INTMSAlign_HiSol reported in Ref.^36^ is based on the software INTMSAlign, which is described in Ref.^35^. As outlined in this Ref.^35^, the software INTMSAlign is available from the corresponding author Prof. Dr. Yasuhisa Asano upon request via E-mail, and furthermore this software is planned to become opened to be used by means of a web site for downloading the software.

### Generation of the mutant library

Site-directed mutagenesis of the plasmid pET22b-OxdB(C)6His was carried out with the QuikChange Lightning Site-directed Mutagenesis Kit (Stratagene, California, USA). A pET22b vector containing the OxdB gene fused to a C-terminal hexahistidine-tag (pET22b-OxdB(C)6His) was used as a dsDNA template in site-directed mutagenesis. The reaction mixture comprised of 0.5 µL of 10 × reaction buffer, 0.2 µL of Quick Solution reagent, 0.2 µL of dNTP mix, 0.2 µL of QuikChange Lightning Enzyme, 125 ng of each the forward and reverse primers (see Supplementary Information, Table S2), 20–30 ng of the plasmid as template DNA, and sterilised water to a final volume of 5 µL. After initial denaturation at 95 °C for 2 min, 18 cycles were repeated as subsequently stated: A denaturing step at 95 °C for 20 s, an annealing step at 50 °C for 10 s, and an elongation step at 68 °C for 3.5 min. The product was treated with 0.2 µL of DpnI at 37 °C for 2 h, and then transformed to chemocompetent cells of *E.* *coli* BL21(DE3).

### Protein expression and purification

Expression and purification were performed according to a slightly modified literature known protocol^32^. A pre-culture of *E.* *coli* BL21(DE3) harbouring pET22b-OxdB(C)6His or its variants was inoculated from a LB-agar-plate and grown for 13 h in test-tubes containing 5 mL LB-medium (Carl Roth GmbH + Co. KG, Karlsruhe, Germany) and 50 µg/mL Carbenicillin (Carl Roth GmbH + Co. KG, Karlsruhe, Germany) at 37 °C at 160 rpm rotary shaking. Subsequently, a main culture of 350 mL Terrific-broth medium (Carl Roth GmbH + Co. KG, Karlsruhe, Germany) containing 50 µg/mL Carbenicillin in a 500 mL Erlenmeyer flask was inoculated with 1 mL of the pre-culture. The culture was incubated for approximately 7 h at 30 °C and 150 rpm rotary shaking until the OD_600_ reached approximately 0.8 and induced with 1 mM isopropyl-*β*-d-thiogalactopyranoside (Gerbu Biotechnik GmbH, Heidelberg, Germany). After induction, the incubation conditions were changed to 26 °C and 120 rpm rotary shaking. After 21 h, the cells were harvested by centrifugation (4000×*g*, 4 °C, 15 min) and washed twice with equilibration buffer (Tris–HCl buffer (20 mM, pH 8.0) containing 300 mM sodium chloride and 10 mM imidazole).

The cells from two cultures (~ 1.0 g) were resuspended with equilibration buffer (1:5 m,*v*) disrupted with sonotrode, and the cell debris and insoluble protein was removed via centrifugation (18,000×*g*, 4 °C, 60 min). The obtained cell-free extract was applied to a column containing TALON Metal Affinity Resin (Takara Biotechnology (DALIAN) Co., LTD., Dalian, PR China) (4.0 mL) equilibrated with wash buffer. After the column had been washed thoroughly (10 *cv*) with the same buffer, the enzyme was eluted with equilibration buffer (Tris–HCl buffer (20 mM, pH 8.0) containing 300 mM sodium chloride and 150 mM imidazole). The purification step was performed at < 20 °C. The combined active fractions were dialysed in Amicon Ultra centrifugal filters (Merck KGaA, Darmstadt, Germany) towards Tris–HCl buffer (20 mM, pH 7.0).

### Activity assay

The activity was determined in modified protocol according to literature known methods^32^. The assay solution contained 437.5 µL potassium phosphate buffer (50 mM, pH 7.0), 12.5 µL of *Z*-PAOx (200 mM solution in dimethyl sulfoxide, final concentration 5.0 mM), 50.0 µL of purified OxdB(C)6His or its variants (0.15 mg mL^−1^; 3.75 µM), in a total volume of 500 µL. The reaction was carried out for 1 min at 30 °C with shaking at 900 rpm. Simultaneous addition of 400 µL of acetonitrile (ACN) and 100 µL of hydrochloric acid (0.1 M) stopped the reaction. After centrifugation (21,500×*g*, 10 min, 4 °C), the supernatant was transferred into HPLC vials and the conversion to PAN was measured by reversed phase-HPLC (Jasco, Nova Scotia, Canada) in comparison to a calibration curve. Measurements were conducted on a Nucleodur C18 HTec column (Macherey–Nagel, Düren, Germany) at 40 °C isocratic with water/ACN (70:30 *v*/*﻿v*) as mobile phase and UV detection at 210 nm. Assays were conducted in triplicates for each variant.

### Mass spectrometry

Accurate nanoESI measurements of proteins were performed using a Q-IMS-TOF mass spectrometer Synapt G2Si (Waters GmbH, Manchester, UK) in resolution mode, interfaced to nano-ESI ion source. Nitrogen served as both the nebuliser gas and the dry gas for nanoESI. Nitrogen is generated by a nitrogen generator NGM 11. Samples (10 µM protein) were dissolved in 0.1 M ammonium acetate (pH 7.0) for native state measurements or 50% acetonitrile containing 0.1% formic acid for measurements under denaturing conditions. The samples were filled into *in-house* made ESI-emitters. The mass axis was externally calibrated with Agilent tune mix as calibration standard. Scan accumulation and data processing (baseline substraction, smoothing and centroidation) was performed with MassLynx 4.1 (Waters GmbH, Manchester, UK) on a PC Workstation. The spectra shown here were generated by the accumulation and averaging of several single spectra. Determination of the accurate mass of protoheme IX and protein masses were performed using centroided data. The protein masses were determined using the software ESIprot from Winkler^49^.

### Substrate and reference compound synthesis

#### Synthesis of Z-phenylacetaldoxime

Hydroxylamine hydrochloride (10.3 g, 148 mmol) and sodium carbonate (17.3 g, 163 mmol) were dissolved in H_2_O at room temperature. Phenylacetaldehyde (12.04 g, 100 mmol) was added and the reaction mixture was stirred vigorously for 18 h until complete conversion according to TLC analysis (cyclohexane/ethyl acetate) was achieved. The solution was extracted thrice with ethyl acetate (1:1 *v*/*v*), the combined organic phases were washed with brine (1:3 *v*/*﻿v*) and dried over MgSO_4_. The solvent was removed *in* *vacuo* gave a crude product, which was purified by recrystallisation (diethyl ether/di*iso*-propylether, − 80 °C) yielding *Z*-phenylacetaldoxime (5.60 g, 41.4 mmol, 41%) with > 99% isomerical purity (according to ^1^H-NMR) as colourless crystals.

^1^H-NMR (500 MHz, CD_2_Cl_2_): δ [ppm] = 8.88 (s, 1H, CH = NO*H*), 7.37–7.30 (m, 2H,Ph*H*), 7.29–7.22 (m, 3H, Ph*H*), 6.91 (t, ^*3*^*J* = 5.4 Hz, 1H, C*H* = NOH), 3.74 (d, ^*3*^*J* = 5.3 Hz, 2H, Ph-C*H*_*2*_). ^13^C-NMR (500 MHz, CD_2_Cl_2_): δ [ppm] = 151.2, 129.1, 129.1, 129.0, 126.9, 31.9. RP-HPLC: *Macherey–Nagel* Nucleodur C_18_ HTec, water/ACN 70:30, 1.0 mL min^–1^, 40 °C, 210 nm, R_t1_ = 7.54 min, R_t2_ = 8.82 min. The analytical data corresponds with the literature^50^.

#### *Synthesisofphenylacetonitrile*

The synthesis was carried out in analogy to Ma et al.^51^. Copper(II) acetate (8.0 mg, 44 µmol) was dissolved in ACN at room temperature. *Z*-PAOx (51 mg, 380 µmol) was added and the solution was heated to reflux for 90 min. Complete conversion was determined via TLC (cyclohexane/ethyl acetate) and the solvent was removed in vacuo. The crude product was suspended in cyclohexane/ethyl acetate (2:1 *v*/*﻿v*) and filtered over a short plug of silica. Removement of the solvent in vacuo yielded phenylacetonitrile (30 mg, 250 µmol, 66%) as a yellow oil.

^1^H-NMR (126 MHz, CDCl_3_): 7.40–7.37 (m, 2H, Ar*H*), 7.35–7.33 (m, 3H, Ar*H*), 3.76 (s, 2H, N≡C–C*H*_*2*_). ^13^C-NMR (126 MHz, CDCl_3_): δ [ppm] = 130.1, 129.3, 128.2, 128.1, 118.0, 23.8. RP-HPLC: *Macherey–Nagel* Nucleodur C_18_ HTec, water/acetonitrile 70:30, 1.0 mL min^–1^, 40 °C, 210 nm, R_t_ = 12.1 min. The analytical data corresponds with the literature^25^.

## Supplementary Information

Supplementary Information 1.
