# Supplementary material for: Protein engineering of the aldoxime dehydratase from Bacillus sp. OxB-1 based on a rational sequence alignment approach
Source: Sci Rep. 2021 Jul 12;11:14316. doi: 10.1038/s41598-021-92749-0 (PMC8275659; doi:10.1038/s41598-021-92749-0)
Supplement: Supplementary file 1 — Supplementary Information 1. [file 41598_2021_92749_MOESM1_ESM.pdf]

Supplementary Information

Protein engineering of the aldoxime dehydratase from *Bacillus* sp. OxB-1 based on a rational sequence alignment approach

Keiko Oike[1], Jens Sproß[1], Daisuke Matsui[2], Yasuhisa Asano[2], Harald Gröger[1,*]

[1]Chair of Industrial Organic Chemistry and Biotechnology, Faculty of Chemistry, Bielefeld University, Universitätsstraße 25, Bielefeld 33615, Germany

[2]Biotechnology Research Center and Department of Biotechnology, Toyama Prefectural University, 5180 Kurokawa, Imizu, Toyama, 939-0398, Japan

1. General information

All enzymes and cells were handled, unless otherwise stated at 4 °C. The variants (OxdB-M1 to OxdB-M20 and Oxd-D1 to Oxd-D3 refer to the single point mutations introduced in the main paper (e.g. Table 1)

2. Sequence of OxdB

Gene (359 AS, 41.2 kDa)

ATGAAAAATATGCCGGAAAATCACAATCCACAAGCGAATGCCTGGACTGCCGAATTTCCTCCTGA
AATGAGCTATGTAGTATTTGCGCAGATTGGGATTCAAAGCAAGTCTTTGGATCACGCAGCGGAAC
ATTTGGGAATGATGAAAAAGAGTTTCGATTTGCGGACAGGCCCCAAACATGTGGATCGAGCCTTG
CATCAAGGAGCCGATGGATACCAAGATTCCATCTTTTTAGCCTACTGGGATGAGCCTGAAACATT
TAAATCATGGGTTGCGGATCCTGAAGTACAAAAGTGGTGGTCGGGTAAAAAAATCGATGAAAATA
GTCCAATCGGGTATTGGAGTGAGGTAACGACCATTCCGATTGATCACTTTGAGACTCTTCATTCC
GGAGAAAATTACGATAATGGGGTTTCACACTTTGTACCGATCAAGCATACAGAAGTCCATGAATAT
TGGGGAGCAATGCGCGACCGCATGCCGGTGTCTGCCAGTAGTGATTTGGAAAGCCCCCTTGGC
CTTCAATTACCGGAACCCATTGTCCGGGAGTCTTTCGGAAAACGGCTAAAAGTCACGGCGCCGG
ATAATATTTGCTTGATTCGAACCGCTCAAAATTGGTCTAAATGTGGTAGCGGGGAAAGGGAAACG
TATATAGGACTAGTGGAACCGACCCTCATAAAAGCGAATACGTTTCTTCGTGAAAATGCTAGTGAA
ACAGGCTGTATTAGTTCAAAATTAGTCTATGAACAGACCCATGACGGCGAAATAGTAGATAAATCA
TGTGTCATCGGATATTATCTCTCCATGGGGCATCTTGAACGCTGGACGCATGATCATCCAACACA
TAAAGCGATCTACGGAACCTTTTATGAGATGTTGAAAAGGCATGATTTTAAGACCGAACTTGCTTT
ATGGCACGAGGTTTCGGTGCTTCAATCCAAAGATATCGAGCTTATCTATGTCAACTGCCATCCGA
GTACTGGATTTCTTCCATTCTTTGAAGTGACAGAAATTCAAGAGCCTTTACTGAAAAGCCCTAGCG
TCAGGATCCAGCTCGAGCACCACCACCACCACCACTGAGATCCGGCTGCTAACAAAGCCCGAAA
GAAGTTTTTT

Amino acids
MKNMPENHNPQANAWTAEFPPEMSYVVFAQIGIQSKSLDHAAEHLGMMKKSFDLRTGPKHVDRALH
QGADGYQDSIFLAYWDEPETFKSWVADPEVQKWWSGKKIDENSPIGYWSEVTTIPIDHFETLHSGEN
YDNGVSHFVPIKHTEVHEYWGAMRDRMPVSASSDLESPLGLQLPEPIVRESFGKRLKVTAPDNICLIR
TAQNWSKCGSGERETYIGLVEPTLIKANTFLRENASETGCISSKLVYEQTHDGEIVDKSCVIGYYLSMG
HLERWTHDHPTHKAIYGTFYEMLKRHDFKTELALWHEVSVLQSKDIELIYVNCHPSTGFLPFFEVTEIQ
EPLLKSPSVRIQ**LEHHHHHH**

2. Result sheet of INTMSAlign_HiSol

The program INTMSAlign_HiSol was applied to the sequence of OxdB according to literature known methods as mentioned in the main work.[1]

Position	Amino acid (OxdB)	Conservation [%]																				HiSol score
		A	C	D	E	F	G	H	I	K	L	M	N	P	Q	R	S	T	V	W	Y	none	
319	I	1.7	0.1	2.4	90.0	0.2	0.6	0.2	0	0.4	0.1	0.8	0	0.1	0.3	0.5	0.3	0.2	0.4	0.1	0.1	1.7	2.656
226	I	0.6	1.3	3.9	2.9	0.2	0.4	5.3	3.5	7.9	1.1	6.2	1.1	0.1	26.5	30.0	1.4	2.3	4.3	0	0	1.1	2.411
220	L	4.3	0	20.7	39.7	0	0.6	0.9	0.2	5.8	0	0.6	8.7	0.2	3.0	1.6	2.5	9.9	0.3	0	0	1.2	2.281
149	V	0.1	0	0.6	65.6	1.1	0.1	0.9	0.4	7	7.9	0.5	1.8	0.1	1.1	1.4	0	4.1	1.8	0.3	3.0	2.3	2.278
105	I	12.7	0.3	8.2	6.9	0.3	2.3	0.6	0	2.5	2.5	0.1	0.7	7.4	2.3	32.6	2.1	3.1	1.3	0	0	14.3	2.252
267	L	6.7	1.0	14.9	2.0	0	1.2	3.4	0.6	9.5	4.9	1.6	0.8	0.2	3.3	40.0	2.8	5.8	0.5	0	0.2	0.7	2.226
316	I	14.0	1.0	0.4	0.1	0.4	0.2	1.0	4.3	0	1.5	1.7	0.6	0.8	67.9	0.3	0.4	1.1	2.2	0	0	2.2	2.175
180	I	13.9	0.4	12.3	5.2	1.2	6.1	0.8	0.3	2.2	2.0	0.5	3.4	5.1	2.9	6.1	12.4	7.3	3.6	0	0.3	14.1	1.890
65	L	5.9	1.6	1.9	2.6	2.9	1.2	24.2	1.0	0.4	0	0.9	0.5	0.2	12	14.1	11.2	3.6	6.5	0.5	5.6	3.5	1.880
262	V	3.8	0.3	7.7	2.3	0.1	43.8	0	0.1	0.6	0.1	0.1	23.4	0	4.7	0.1	2.3	2.0	7.1	0	0.4	1.2	1.852
317	E	2.8	0.3	5.3	1.4	11.9	0.5	4.5	3.4	0.2	17.8	1.0	0.6	0.7	2.9	7.2	7.6	5.1	13.7	2.1	9.2	2.1	-1.452
190	K	0.7	0.7	0.1	0.7	2.3	1.0	0.7	8.0	0	4.7	1.6	0.1	0.1	1.1	34.5	1.5	5.3	34.2	0.4	0.3	2.0	-1.471
135	D	2.9	0.1	0.8	1.0	8.7	4.9	0.2	1.0	1.9	35.0	2.1	2.0	25.3	1.9	3.8	1.2	0.8	1.5	0.3	0.7	4.0	-1.496
67	Q	3.6	0.1	5.0	4.9	0.3	0.5	1.4	8.9	1.1	8.1	0.4	1.6	1.5	0	1.7	0.7	32.2	24.2	0	0.5	3.5	-1.533
49	K	15.0	0.3	4.5	3.2	1.5	1.7	1.6	5.0	0.9	6.9	1.1	1.8	0.7	4.2	9.7	3.7	7.1	24.2	1.1	0.1	5.6	-1.582
63	R	2.4	0.2	0.3	2.2	8.5	0.3	3.2	5.1	0.3	34.6	2.2	0.3	1.1	1.3	15.8	6.3	4.0	5.2	2.6	0.3	4.0	-1.882
296	H	0.8	0.1	0.2	0.6	13.5	5.3	0	0.7	0.4	56.7	4.3	0.4	0.1	1.2	0.3	0.4	0.5	1.5	0.7	7.4	5.0	-1.943
229	N	0.5	0	0	0	0.1	0	0.1	1.9	0	10.0	80.1	0	0	0	0	0	0.3	6.1	0	0	0.8	-1.974
250	Q	1.0	0.7	0.7	3.3	3.9	0.5	0.4	29.2	0.5	24.0	1.8	0.2	2.0	2.0	2.7	1.4	3.1	19.5	0	0.4	2.7	-2.153
283	K	2.7	0	0.3	0.7	0.3	0.1	1.5	2.4	1.9	57.8	0.3	0	0	0.5	1.3	0.3	0.2	28.8	0	0	1.0	-2.485

Table S1. Output sheet of INTMSAlign_HiSol applied to the sequence of OxdB according to the formula given in chapter 9.2**.** Conservation (similarity) of all amino acid residues in the gene family for the positions, which exhibited the highest absolute HiSol. The suggested mutations are marked in yellow.

3. Utilised primers

The following primers were utilized for introduction of the point mutations into the sequence of Oxds according to a PCR method displayed in the main paper.

Entry	Mutation	Sequence (5' to 3')
1	I319E	Forward: GATATCGAGCTT**GAA**TATGTCAACTGCCATC<br>Reverse: GATGGCAGTTGACATA**TTC**AAGCTCGATATC
2	I226Q	Forward: GACCCTC**CAA**AAAGCGAATACGTTTC<br>Reverse: GAAACGTATTCGCTTT**TTG**GAGGGTC
3	L220E	Forward: CGTATATAGGA**GAA**GTGGAACCGACCCTC<br>Reverse: GAGGGTCGGTTCCAC**TTC**TCCTATATACG
4	V149E	Forward: GTACCGATCAAGCATACAGAA**GAG**CATGAATATTG<br>Reverse: CAATATTCATG**CTC**TTCTGTATGCTTGATCGGTAC
5	I105R	Forward: GTCGGGTAAAAAA**AGA**GATGAAAATAGTCCAATC<br>Reverse: GATTGGACTATTTTCATC**TCT**TTTTTTACCCGAC
6	L267R	Forward: GTGTCATCGGATATTAT**CGC**TCCATGG<br>Reverse: CCATGGA**GCG**ATAATATCCGATGACAC
7	I316Q	Forward: CAATCCAAAGAT**CAG**GAGCTTATCTATGTCAAC<br>Reverse: GTTGACATAGATAAGCTC**CTG**ATCTTTGGATTG
8	I180A	Forward: CTTCAATTACCGGAACCC**GCT**GTCC<br>Reverse: GGAC**AGC**GGGTTCCGGTAATTGAAG
9	L65H	Forward: CATGTGGATCGAGCC**CAC**CATCAAG<br>Reverse: CTTGATG**GTG**GGCTCGATCCACATG
10	V262G	Forward: CATGT**GGC**ATCGGATATTATCTCTCCATG<br>Reverse: CATGGAGAGATAATATCCGAT**GCC**ACATG
11	E317L	Forward: CCAAAGATATC**CTG**CTTATCTATGTCAACTGCC<br>Reverse: GGCAGTTGACATAGATAAG**CAG**GATATCTTTGG
12	K190R	Forward: CTTTCGGAAAACGGCTA**AGA**GTCACG<br>Reverse: CGTGAC**TCT**TAGCCGTTTTCCGAAAG
13	D135L	Forward: GAGAAAATTAC**CTA**AATGGGGTTTCACACTTTGTAC<br>Reverse: GTACAAAGTGTGAAACCCCATT**TAG**GTAATTTTCTC
14	Q67T	Forward: CCTTGCAT**ACA**GGAGCCGATGGATAC<br>Reverse: GTATCCATCGGCTCC**TGT**ATGCAAGG
15	K49V	Forward: CATTTGGGAATGATG**GTA**AAGAGTTTCGATTTG<br>Reverse: CAAATCGAAACTCTT**TAC**CATCATTCCCAAATG
16	R63L	Forward: CAAACATGTGGAT**CTA**GCCTTGCATCAAG<br>Reverse: CTTGATGCAAGGC**TAG**ATCCACATGTTTG
17	H296L	Forward: GTTGAAAAGG**CTT**GATTTTAAGACCGAACTTG<br>Reverse: CAAGTTCGGTCTTAAAATC**AAG**CCTTTTCAAC
18	N229M	Forward: CTCATAAAAGCG**ATG**ACGTTTCTTCGTG<br>Reverse: CACGAAGAAACGT**CAT**CGCTTTTATGAG
19	Q250I	Forward: CTATGAA**ATC**ACCCATGACGGCGAAATAG<br>Reverse: CTATTTCGCCGTCATGGGT**GAT**TTCATAG
20	K283L	Forward: GATCATCCAACACAT**TTA**GCGATCTACGG<br>Reverse: CCGTAGATCGC**TAA**ATGTGTTGGATGATC

Table S2. Primers used for the introduction of single mutations in the sequence of OxdB *via* PCR with wild-type or mutated pET22b-OxdB(C)6His as templete DNA.

4. Experiments with whole-cell catalysts

Expression and purification were performed according to a slightly modified literature known protocol.[2] A preculture of *E. coli* BL21(DE3) harbouring pET22b-OxdB(C)6His or its variants was inoculated from a LB-agar-plate and grown for 13 hours in test-tubes containing 5.0 mL LB-medium and 50 µg·mL^{-1} Carbenicillin at 37 °C at 160 rpm rotary shaking. Subsequently, a main culture of 70 mL Terrific-broth medium containing 50 µg·mL^{-1} Carbenicillin in a 100 mL Erlenmeyer flask (for whole-cell activity screening: 70 mL medium in 100 mL flask) was inoculated with 700 mL of the preculture. The culture was incubated for approximately 6 hours at 30 °C and 150 rpm rotary shaking and induced with IPTG (1.0 mM final concentration). After induction, the incubation conditions were changed to 26 °C and 120 rpm rotary shaking. After 21 hours, the cells were harvested by centrifugation (4,000 × *g*, 4 °C, 15 min) and washed twice with potassium phosphate buffer (50 mM, pH 7.0). After repeated centrifugation (4,000 × *g*, 4 °C, 15 min), the cells were resuspended in 5.0 vol% of the initial medium volume KPB (50 mM, pH 7.0), overlaid with argon and stored at 4 °C as a resting cells suspension.

The activity assay was carried out according to a literature known method. [3] The standard assay solution contained 387.5 µL of KPB (50 mM, pH 7.0), 12.5 µL of a solution of Z-PAOx in DMSO (200 mM, final concentration: 5.0and mM) and 100 µL of diluted resting cells suspension (2.0 µL resting cells suspension and 98 µL water) in total volume of 500 µL. The reaction was carried out for 1 minute at 30 °C with shaking at 800 rpm and terminated by the addition of 400 µL of acetonitrile and 100 µL of 0.1 M HCl. After centrifugation (21,500 × *g*, 15 min, 4 °C), the supernatant was transferred into HPLC vials and the conversion of the reaction was measured with RP-HPLC in comparison to a calibration curve. Measurements were conducted on a Macherey-Nagel Nucleodur C18 HTec column at 40 °C with acetonitrile/water (30:70 *v*,*v*) as mobile phase and UV detection at 210 nm.

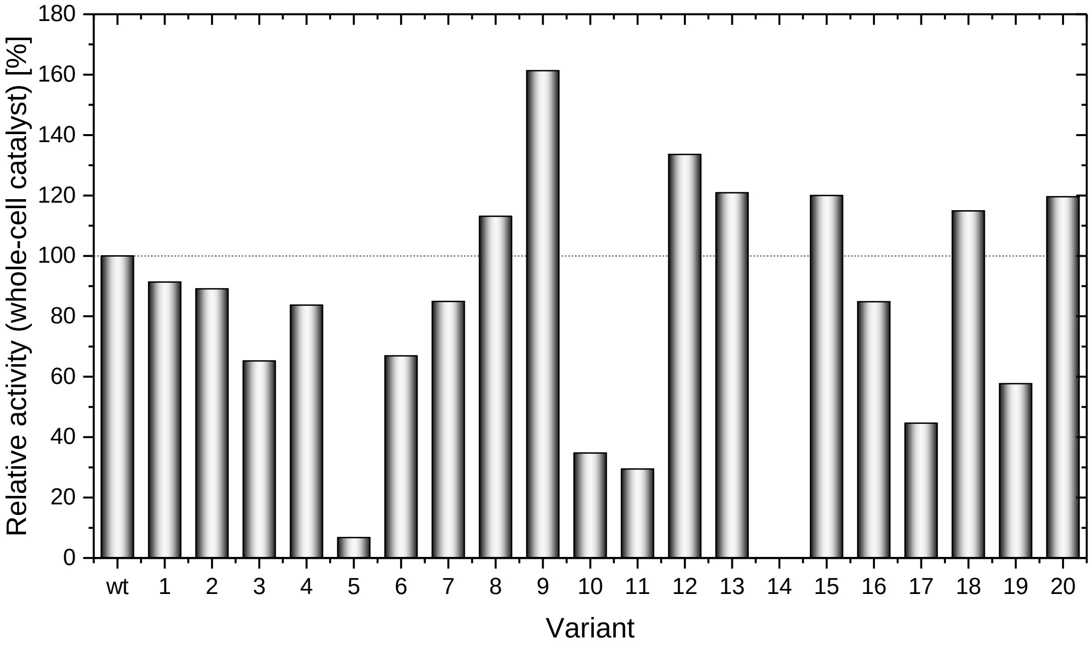

Figure S1. Relative activities of the whole-cell catalysts expressing OxdB variants compared to the wild-type enzyme (0.820 U mg_{bww}^{-1}).

The OxdB-M14 variant was constructed again after the whole-cell catalyst assay and was active in purified form. A test with whole-cell was not carried out again.

5. Electrophoresis of purified variants OxdB variants

Sodium dodecylsulfate polyacrylamide gelelectrophoreis was carried out according to the method by Laemmli. [4] 2.5 µg of the purified protein was loaded on the gels.

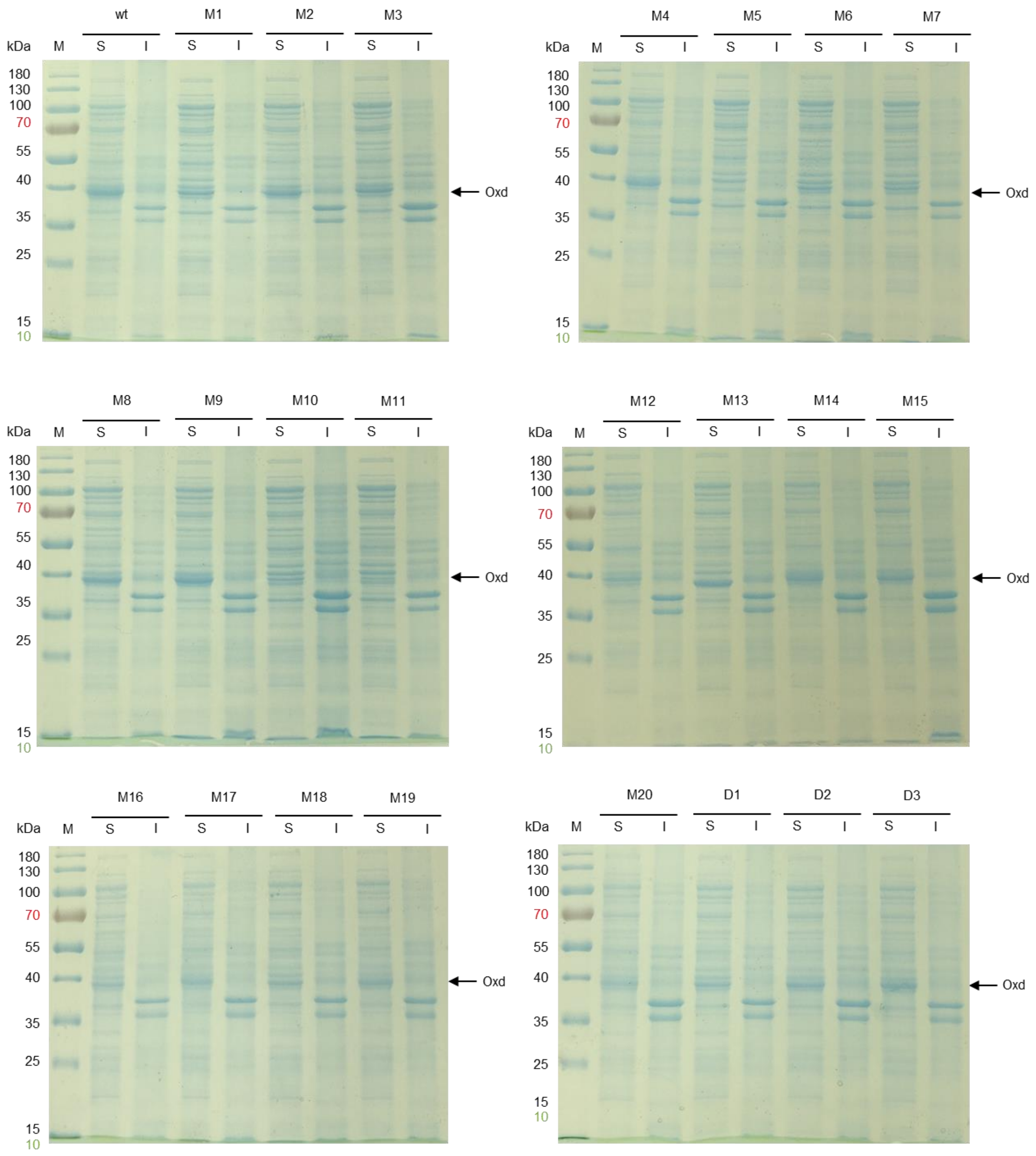

Figure S2. SDS-PAGE analysis of the soluble (S) and insoluble fraction (I) of the expression of wild-type OxdB and the constructed variants.

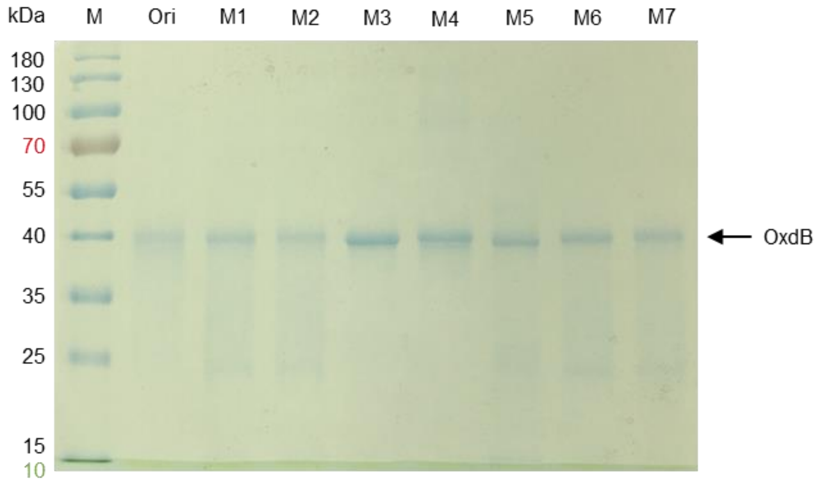

Figure S3. SDS-PAGE of OxdB mutants purified *via* TALON® affinity chromatography. The lanes refer to the corresponding variants (see Table S1).

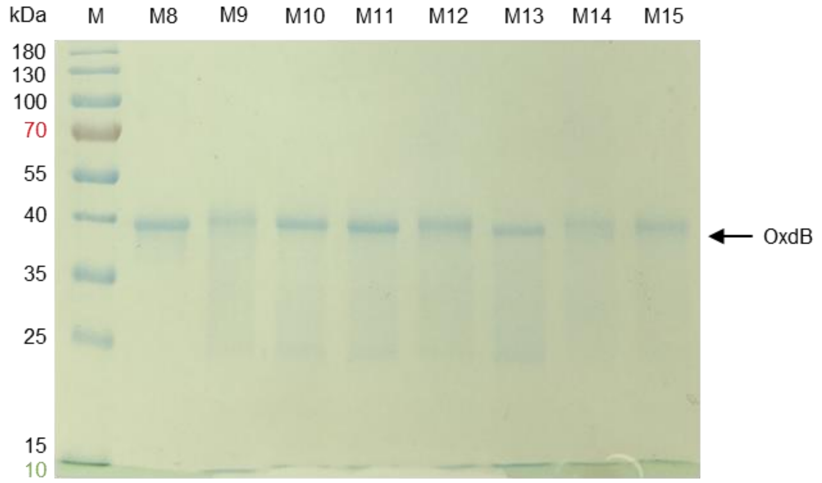

Figure S4. SDS-PAGE of OxdB mutants purified via TALON® affinity chromatography. The lanes refer to the corresponding variants (see Table S1).

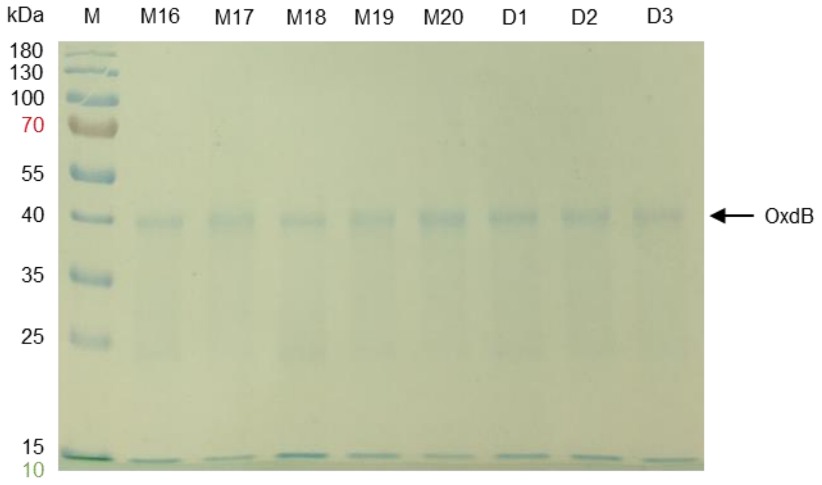

Figure S5. SDS-PAGE of OxdB mutants purified *via* TALON® affinity chromatography. The lanes refer to the corresponding variants (see Table S1).

5. Sequence alignment

```
OxdA     1 MESAIDTHLKCPRTLSRRVPEEYQPPFPMWVARADEQLQQVVMGYLGVQYRGEAQREAAL  60
OxdB     1 MKNMPENHNPQANAWTAEFPPEMSYVVFAQIGIQSKSLDHAAEHLGMMKKSFDLRTGPKH  60
OxdRE    1 MESAIGEHLQCPRTLTRRVPDTYTPPFPMWVGRADDALQQVVMGYLGVQFRDEDQRPAAL  60

OxdA    61 QAMRHIVSSFSLPDGPQTHDLTHHTDSSGFDNLMVVGYWKDPAAHCRWLRSAEVNDWWTS 120
OxdB    61 VDRALHQGADGYQDSIFLAYWDEPETFKSWVADPEVQKWWSGKKIDENSPIGYWSEVTTI 120
OxdRE   61 QAMRDIVAGFDLPDGPAHHDLTHHIDNQGYENLIVVGYWKDVSSQHRWSTSTPIASWWES 120

OxdA   121 QDRLGEGLGYFREISAPRAEQFETLYAFQDNLPGVGAVMDSTSGEIEEHGYWGSMRDRFP 180
OxdB   121 PIDHFETLHSGENYDNGVSHFVPIKHTEVHEYWGAMRDRMPVSASSDLESPLGLQLPEPI 180
OxdRE  121 EDRLSDGLGFFREIVAPRAEQFETLYAFQEDLPGVGAVMDGISGEINEHGYWGSMRERFP 180

OxdA   181 ISQTDWMKPTNELQVVAGDPAKGGRVVIMGHDNIALIRSGQDWADAEAEERSLYLDEILP 240
OxdB   181 VRESFGKRLKVTAPDNICLIRTAQNWSKCGSGERETYIGLVEPTLIKANTFLRENASETG 240
OxdRE  181 ISQTDWMQASGELRVIAGDPAVGGRVVVRGHDNIALIRSGQDWADAEADERSLYLDEILP 240

OxdA   241 TLQDGMDFLRDNGQPLGCYSNRFVRNIDLDGNFLDVSYNIGHWRSLEKLERWAESHPTHL 300
OxdB   241 CISSKLVYEQTHDGEIVDKSCVIGYYLSMGHLERWTHDHPTHKAIYGTFYEMLKRHDFKT 300
OxdRE  241 TLQSGMDFLRDNGPAVGCYSNRFVRNIDIDGNFLDLSYNIGHWASLDQLERWSESHPTHL 300

OxdA   301 RIFVTFFRVAAGLKKLRLYHEVSVSDAKSQVFEYINCHPHTGMLRDAVVAPT         352
OxdB   301 ELALWHEVSVLQSKDIELIYVNCHPSTGFLPFFEVTEIQEPLLKSPSVRIQ          351
OxdRE  301 RIFTTFFRVAAGLSKLRLYHEVSVFDAADQLYEYINCHPGTGMLRDAVTIAEH        353
```

Figure S6. Alignment of the sequences of OxdA, OxdB and OxdRE.

6. Mass spectrometry of OxdB variants

Static nanoESI measurements of the purified OxdB (variants) were carried out under denaturing and native conditions according to methods described in the main paper.

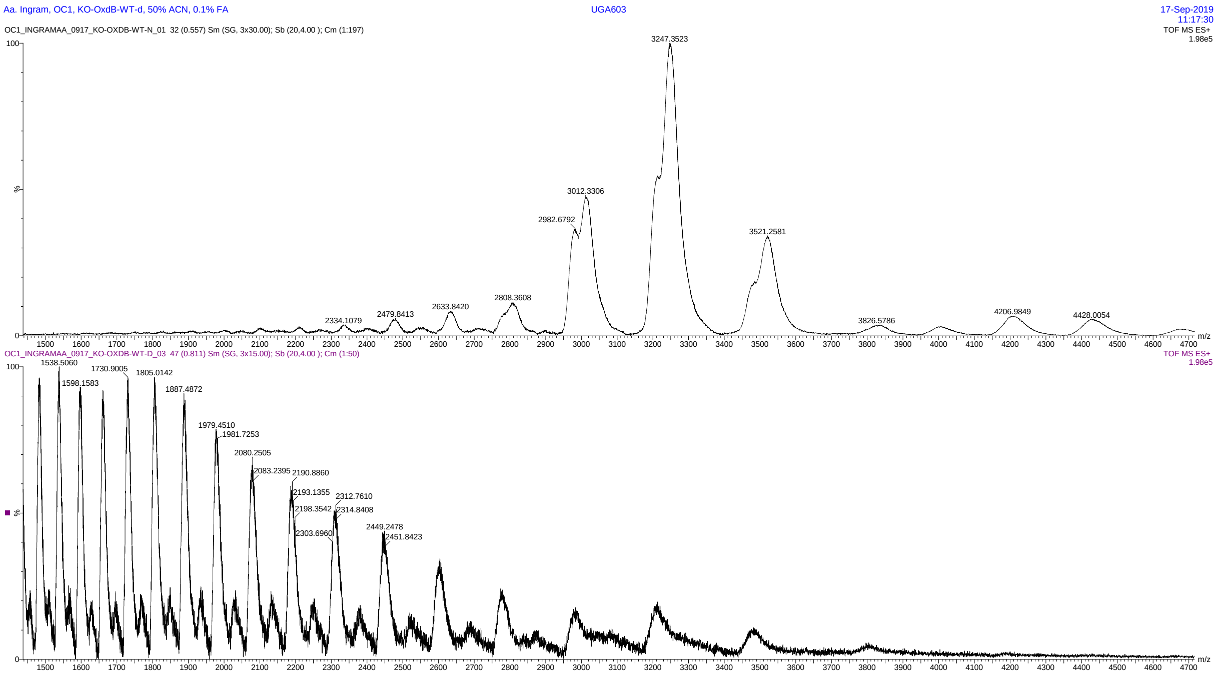

Figure S7. ESI-mass spectrum of native (upper) and denatured (lower) purified wild-type-OxdB.

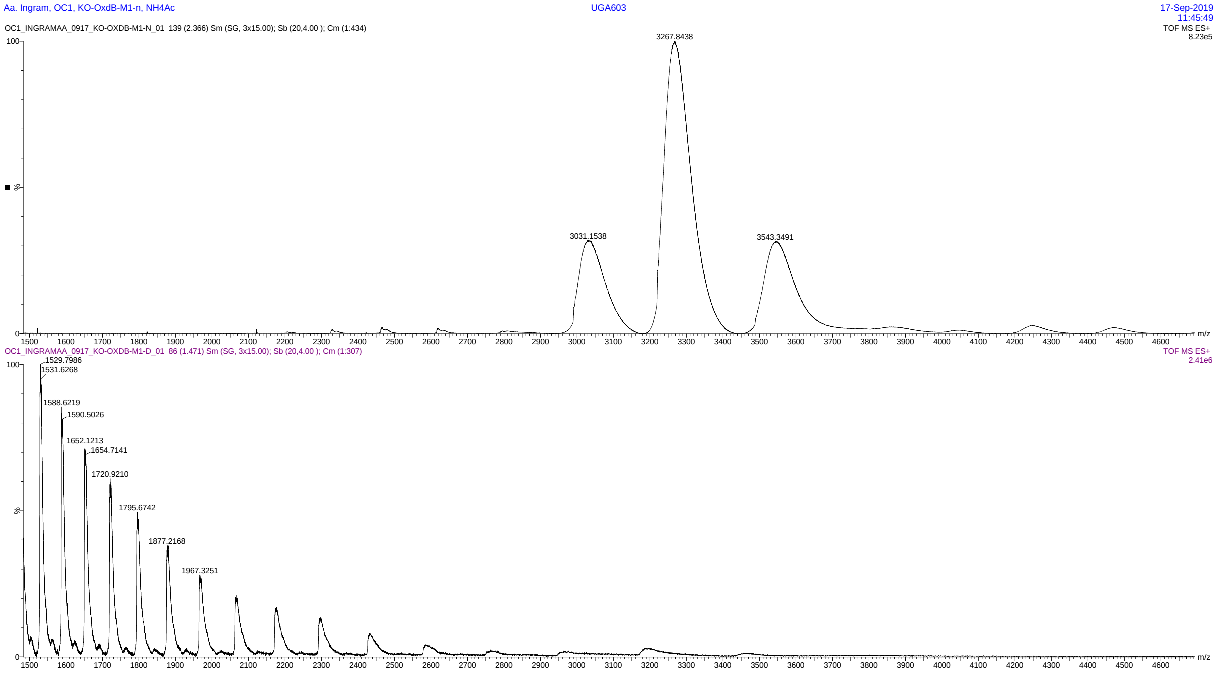

Figure S8. ESI-mass spectrum of native (upper) and denatured (lower) purified OxdB-M1.

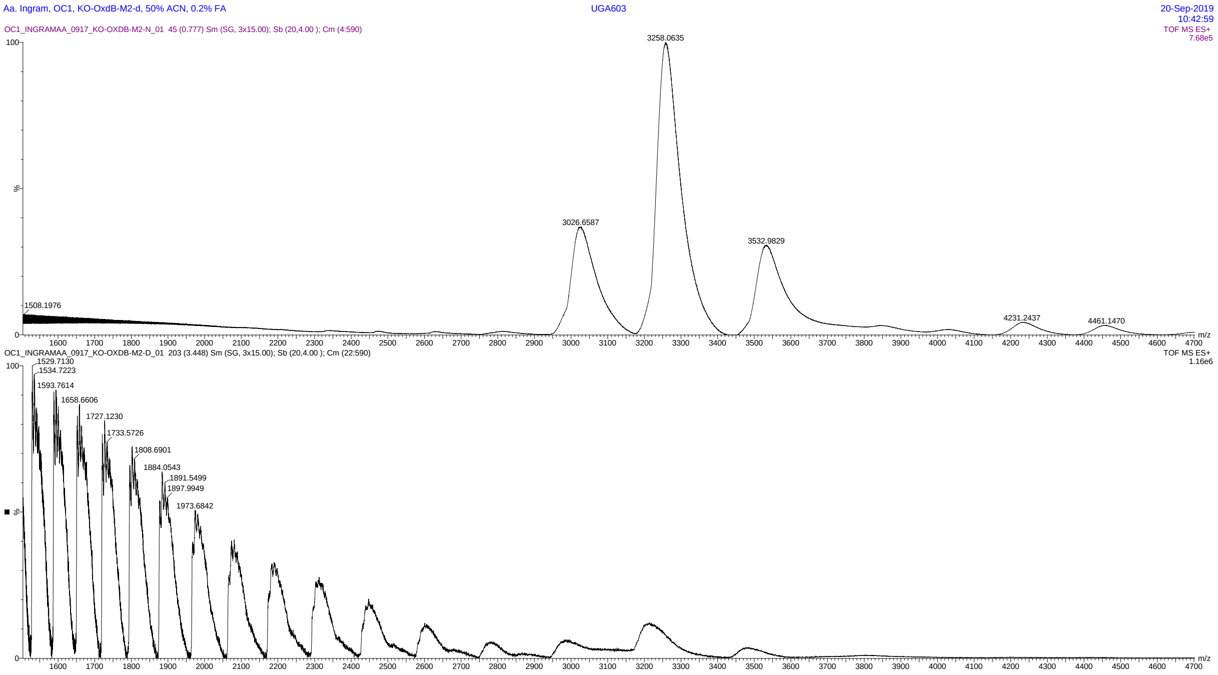

Figure S9. ESI-mass spectrum of native (upper) and denatured (lower) purified OxdB-M2.

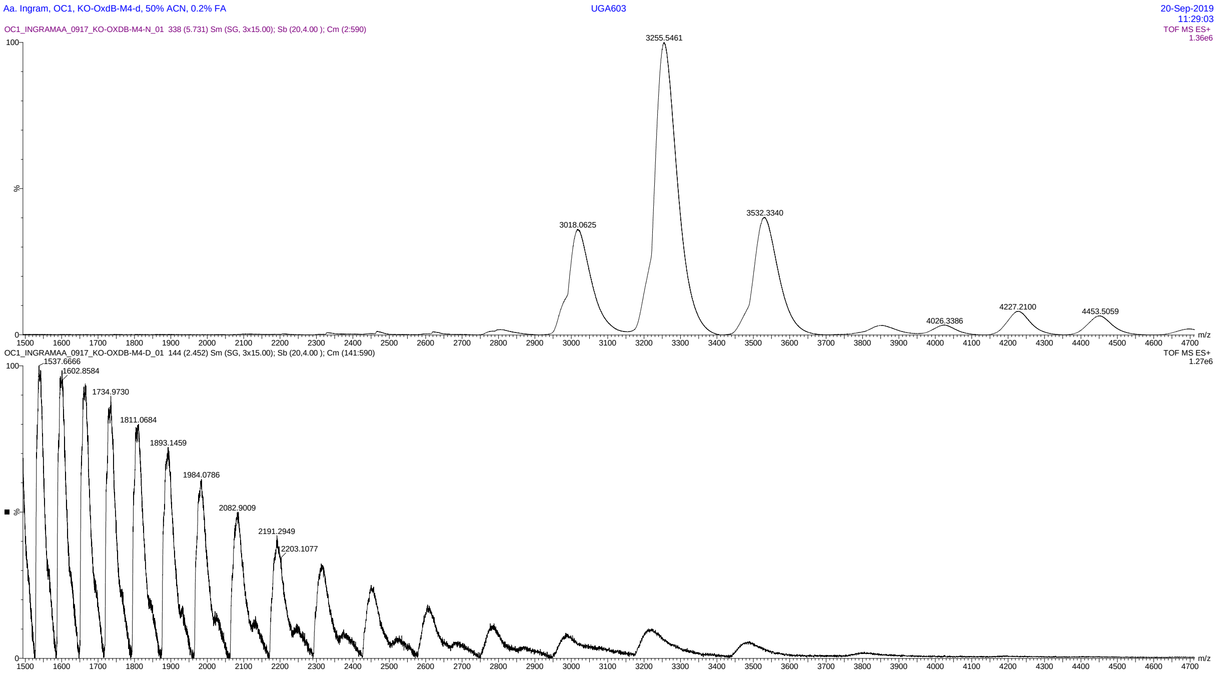

Figure S10. ESI-mass spectrum of native (upper) and denatured (lower) purified OxdB-M4.

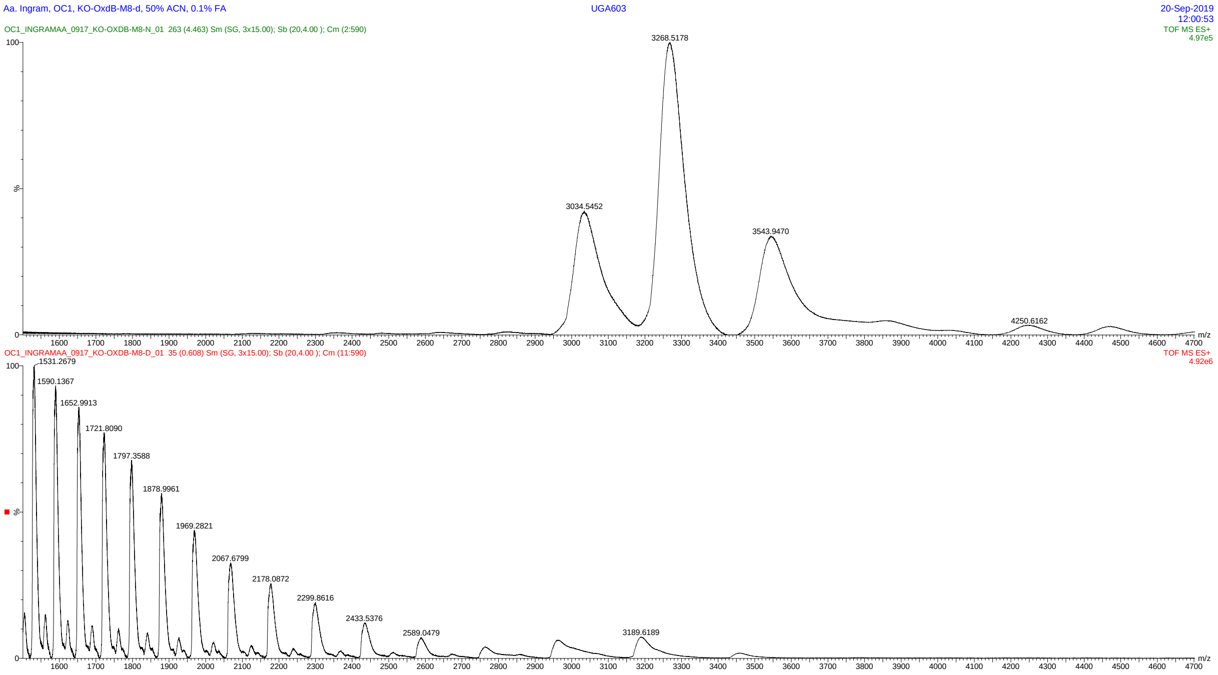

Figure S11. ESI-mass spectrum of native (upper) and denatured (lower) purified OxdB-M8.

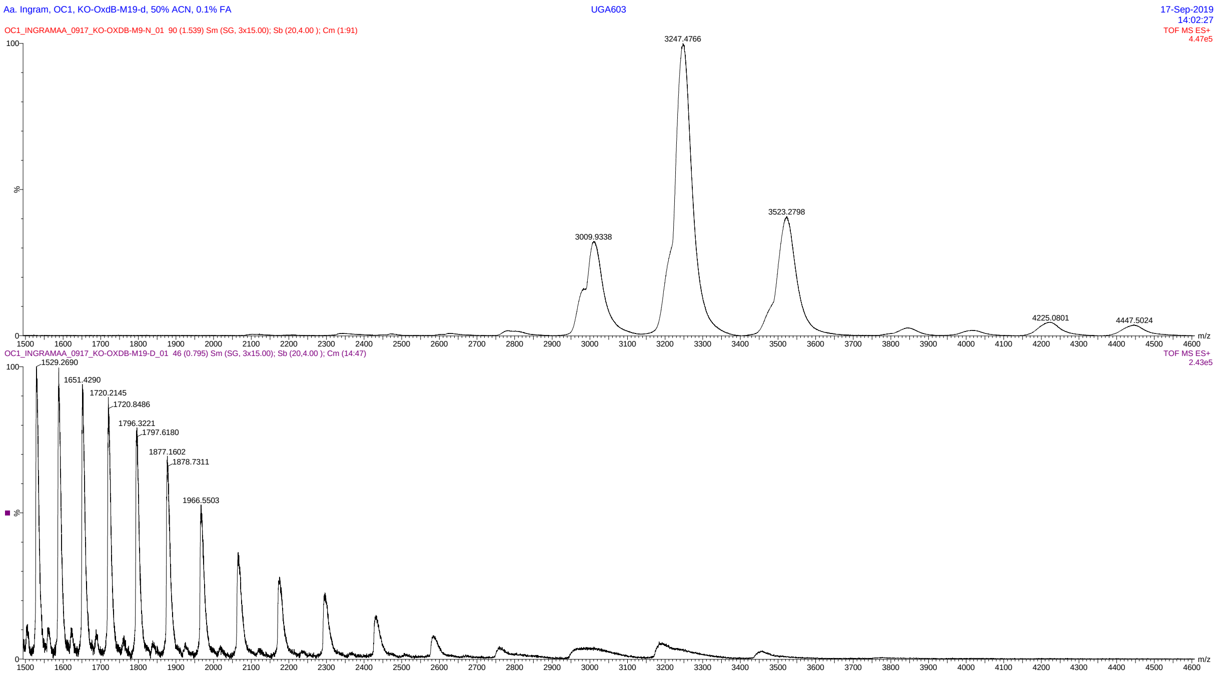

Figure S12. ESI-mass spectrum of native (upper) and denatured (lower) purified OxdB-M9.

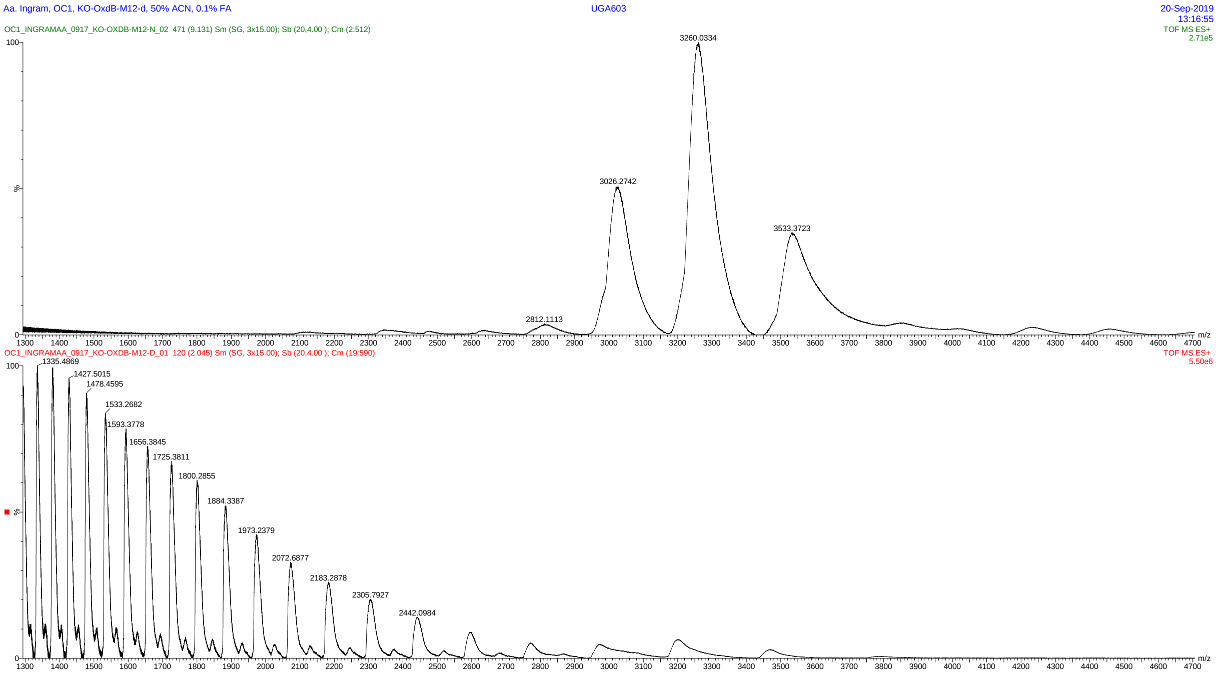

Figure S13. ESI-mass spectrum of native (upper) and denatured (lower) purified OxdB-M12.

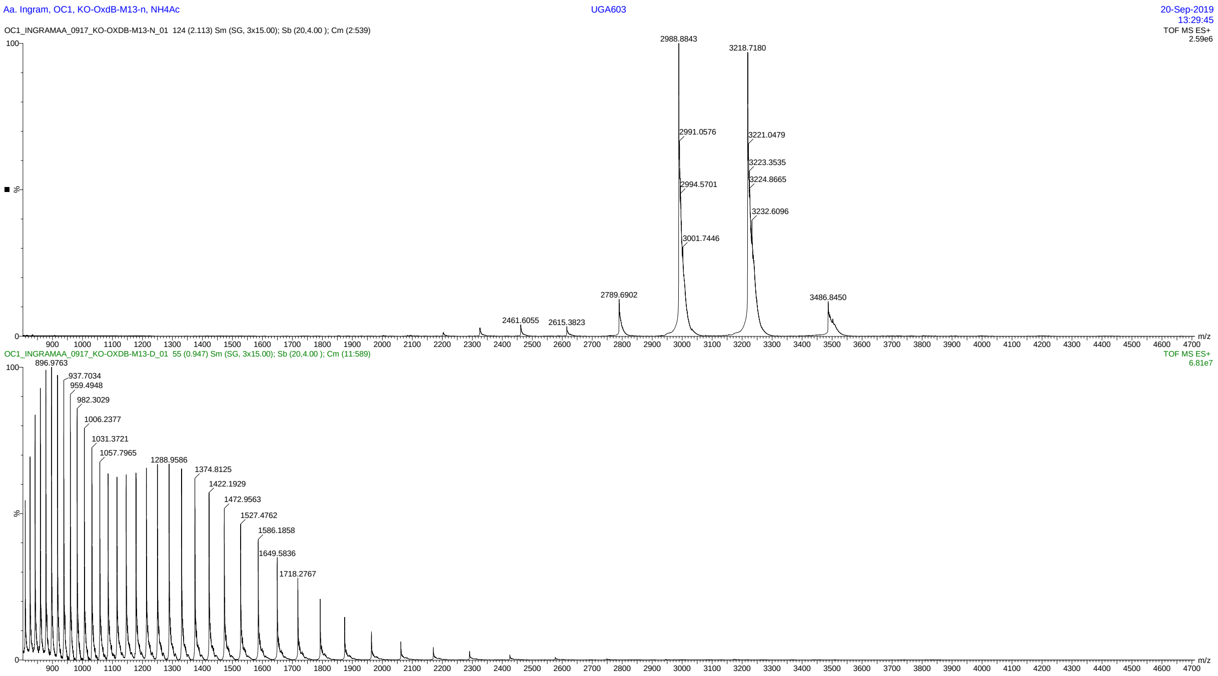

Figure S14. ESI-mass spectrum of native (upper) and denatured (lower) purified OxdB-M13.

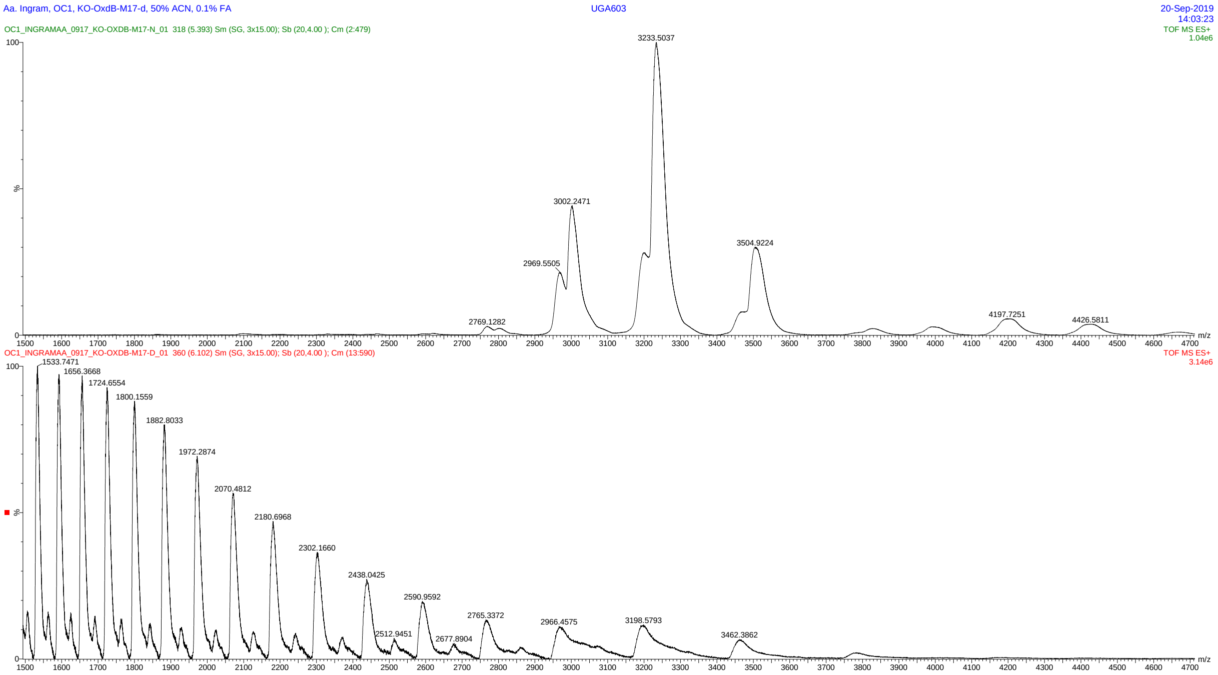

Figure S15. ESI-mass spectrum of native (upper) and denatured (lower) purified OxdB-M17.

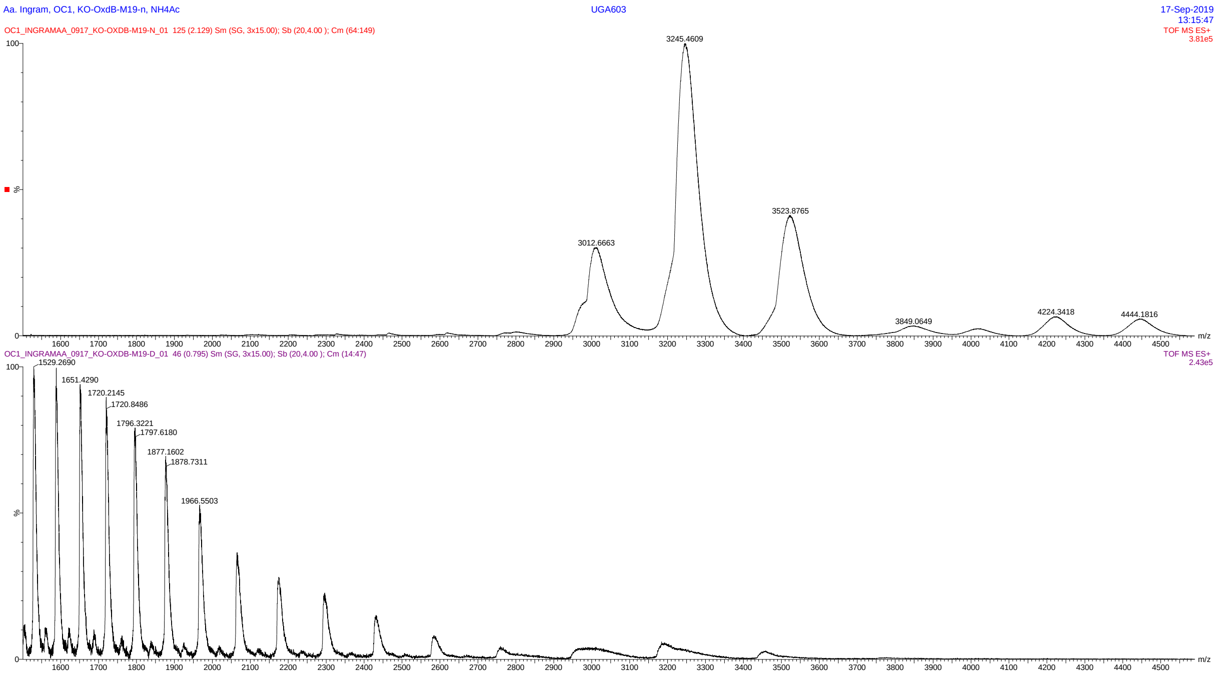

Figure S16. ESI-mass spectrum of native (upper) and denatured (lower) purified OxdB-M19.

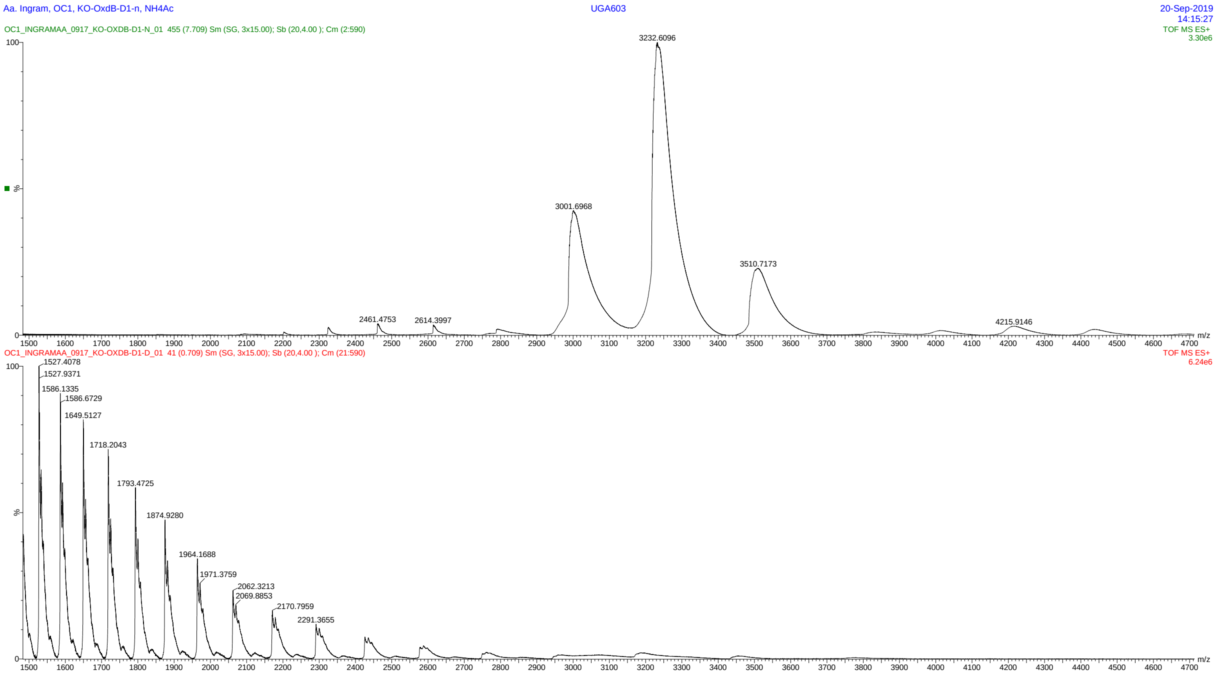

Figure S17. ESI-mass spectrum of native (upper) and denatured (lower) purified OxdB-D1.

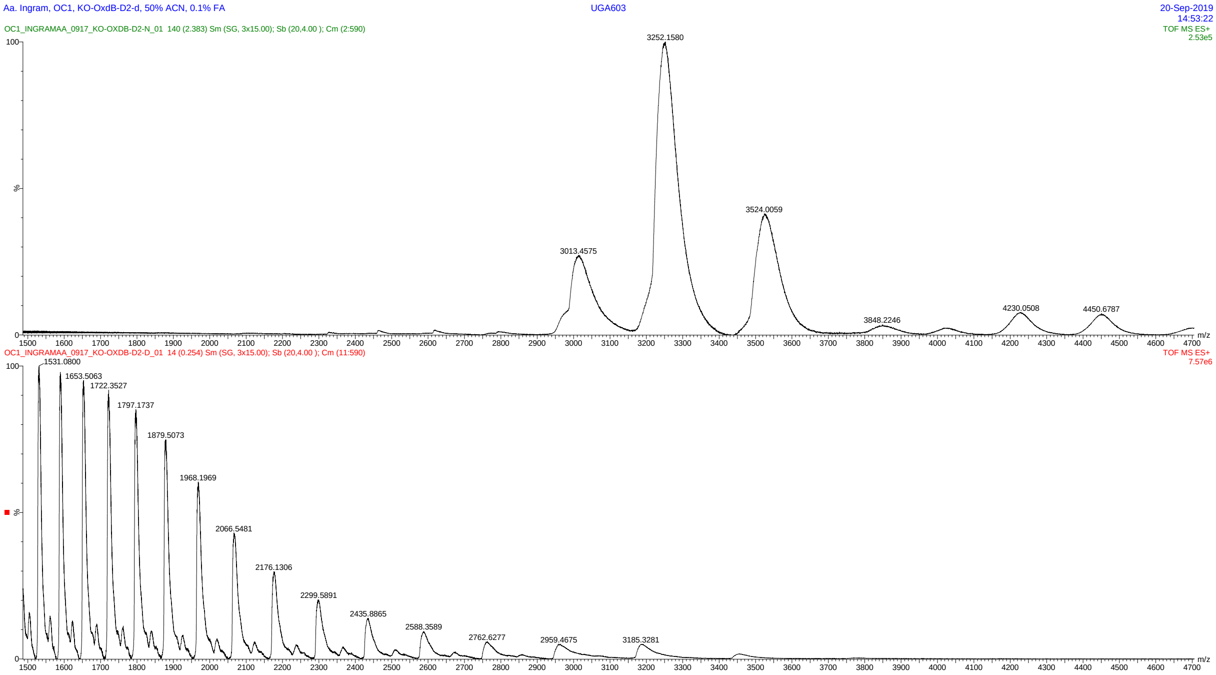

Figure S18. ESI-mass spectrum of native (upper) and denatured (lower) purified OxdB-D2.

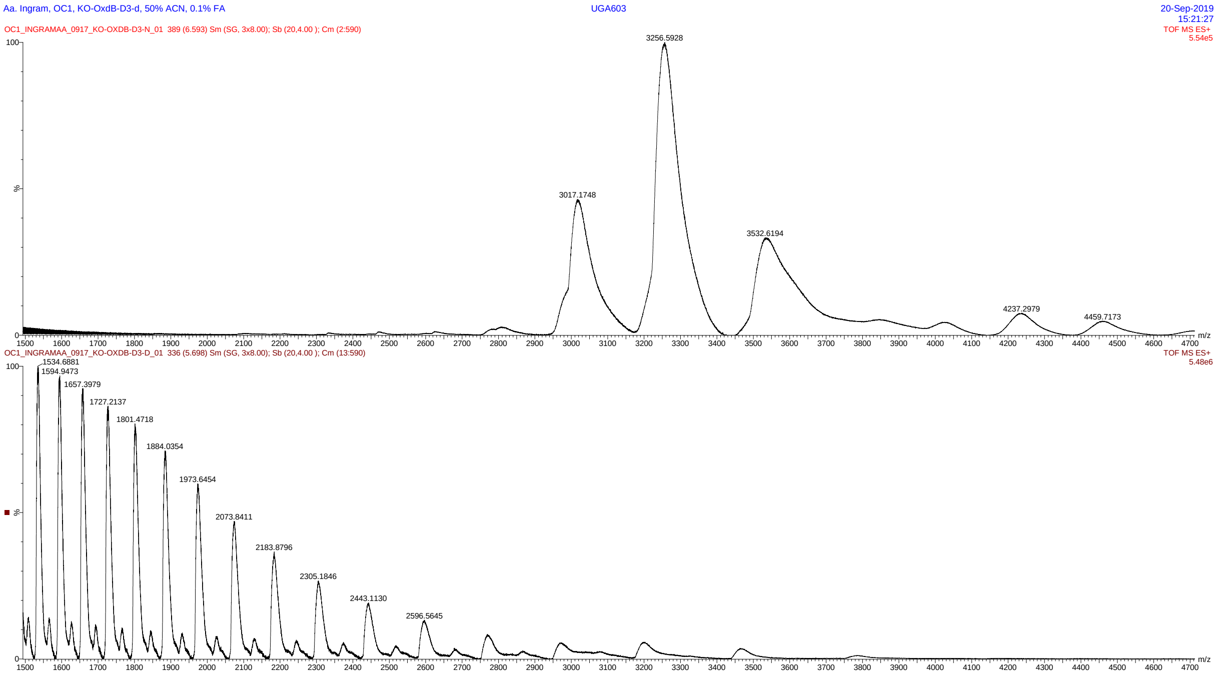

Figure S19. ESI-mass spectrum of native (upper) and denatured (lower) purified OxdB-D3.

Cofactor leaching was discussed as one reason for the uncomplete incorporation of the heme cofactor into Oxds.[5] In our studies, we could find no evidence for cofactor leaching during the purification procedures, which would be visible due to its characteristic deep red colour. Cofactor incorporation up to 98 % was observed in the mass spectra. In addition, the time between activity assay and mass measurement was kept short (4 to 14 days), it was the same between the variants with the highest (OxdB-M1) and the lowest heme content (wild-type OxdB). Precipitation of small amount of heme is only relevant for long incubation times (~4 months).[5]

7. References

1. Matsui, D., Nakano, S., Dadashipour, M. & Asano, Y. Rational identification of aggregation hotspots based on secondary structure and amino acid hydrophobicity. *Sci. Rep.* **7,** 9558 (2017).
2. Miao, Y., Metzner, R. & Asano, Y. Kemp Elimination Catalyzed by Naturally Occurring Aldoxime Dehydratases. *ChemBioChem* **18,** 451–454 (2017).
3. Betke, T., Rommelmann, P., Oike, K., Asano, Y. & Gröger, H. Cyanide-Free and Broadly Applicable Enantioselective Synthetic Platform for Chiral Nitriles through a Biocatalytic Approach. *Angew. Chem. Int. Ed.* **56,** 12361–12366 (2017).
4. Laemmli, U. K. Cleavage of Structural Proteins during the Assembly of the Head of Bacteriophage T4. *Nature* **227,** 680–685 (1979).
5. Kato, Y., Nakamura, K., Sakiyama, H., Mayhew, S. G. & Asano, Y. Novel heme-containing lyase, phenylacetaldoxime dehydratase from Bacillus sp. strain OxB-1: purification, characterization, and molecular cloning of the gene. *Biochemistry* **39,** 800–809 (2000).
